# Improving survival in advanced melanoma patients: a trend analysis from 2013 to 2021

**DOI:** 10.1016/j.eclinm.2024.102485

**Published:** 2024-02-12

**Authors:** Olivier J. van Not, Alfons J.M. van den Eertwegh, John B. Haanen, Christian U. Blank, Maureen J.B. Aarts, Jesper van Breeschoten, Franchette W.P.J. van den Berkmortel, Jan-Willem B. de Groot, Geke A.P. Hospers, Rawa K. Ismail, Ellen Kapiteijn, Manja Bloem, Melissa M. De Meza, Djura Piersma, Rozemarijn S. van Rijn, Marion A.M. Stevense-den Boer, Astrid A.M. van der Veldt, Gerard Vreugdenhil, Marye J. Boers-Sonderen, Willeke A.M. Blokx, Michel W.J.M. Wouters, Karijn P.M. Suijkerbuijk

**Affiliations:** a Scientific Bureau, Dutch Institute for Clinical Auditing, Rijnsburgerweg 10, Leiden 2333AA, the Netherlands; b Department of Medical Oncology, University Medical Center Utrecht, Utrecht University, Heidelberglaan 100, Utrecht 3584CX, the Netherlands; c Department of Medical Oncology, Amsterdam UMC, VU University Medical Center, Cancer Center Amsterdam, De Boelelaan 1118, Amsterdam 1081HZ, the Netherlands; d Department of Molecular Oncology & Immunology, Netherlands Cancer Institute, Plesmanlaan 121, Amsterdam 1066CX, the Netherlands; e Department of Medical Oncology & Immunology, Netherlands Cancer Institute, Plesmanlaan 121, Amsterdam 1066CX, the Netherlands; f Department of Medical Oncology, GROW School for Oncology and Developmental Biology, Maastricht University Medical Centre+, P. Debyelaan 25, Maastricht 6229 HX, the Netherlands; g Department of Medical Oncology, Zuyderland Medical Centre Sittard, Dr. H. van der Hoffplein 1, Sittard-Geleen 6162BG, the Netherlands; h Isala Oncology Center, Isala, Dokter van Heesweg 2, Zwolle 8025AB, the Netherlands; i Department of Medical Oncology, University Medical Centre Groningen, University of Groningen, Hanzeplein 1, Groningen 9713GZ, the Netherlands; j Department of Medical Oncology, Leiden University Medical Centre, Albinusdreef 2, Leiden 2333ZA, the Netherlands; k Department of Biomedical Data Sciences, Leiden University Medical Centre, Einthovenweg 20, Leiden 2333ZC, the Netherlands; l Department of Surgical Oncology, Netherlands Cancer Institute, Plesmanlaan 121, Amsterdam 1066CX, the Netherlands; m Department of Internal Medicine, Medisch Spectrum Twente, Koningsplein 1, Enschede 7512KZ, the Netherlands; n Department of Internal Medicine, Medical Centre Leeuwarden, Henri Dunantweg 2, Leeuwarden 8934AD, the Netherlands; o Department of Internal Medicine, Amphia Hospital, Molengracht 21, Breda 4818CK, the Netherlands; p Department of Medical Oncology and Radiology & Nuclear Medicine, Erasmus Medical Centre, ‘s-Gravendijkwal 230, Rotterdam 3015CE, the Netherlands; q Department of Internal Medicine, Maxima Medical Centre, De Run 4600, Eindhoven 5504DB, the Netherlands; r Department of Medical Oncology, Radboud University Medical Centre, Geert Grooteplein Zuid 10, Nijmegen 6525GA, the Netherlands; s Department of Pathology, University Medical Center Utrecht, Heidelberglaan 100, Utrecht 3584CX, Utrecht University, the Netherlands

**Keywords:** Melanoma, Response, Survival

## Abstract

**Background:**

The prognosis of advanced melanoma patients has significantly improved over the years. We aimed to evaluate the survival per year of diagnosis.

**Methods:**

All systemically treated patients diagnosed with advanced melanoma from 2013 to 2021 were included from the Dutch Melanoma Treatment Registry. Baseline characteristics and overall survival (OS) were compared between the different years of diagnosis. A multivariable Cox proportional hazards model was used to estimate the association between year of diagnosis and OS.

**Findings:**

For this cohort study, we included 6260 systemically treated advanced melanoma patients. At baseline, there was an increase over the years in age, the percentage of patients with an ECOG PS ≥ 2, with brain metastases, and a synchronous diagnosis of primary and unresectable melanoma. Median OS increased from 11.2 months (95% CI 10.0–12.4) for patients diagnosed in 2013 to 32.0 months (95% CI 26.6–36.7) for patients diagnosed in 2019. Median OS was remarkably lower for patients diagnosed in 2020 (26.6 months; 95% CI 23.9–35.1) and 2021 (24.0 months; 95% CI 20.4-NR). Patients diagnosed in 2020 and 2021 had a higher hazard of death compared to patients diagnosed in 2019, although this was not significant. The multivariable Cox regression showed a lower hazard of death for the years of diagnosis after 2013. In contrast, patients diagnosed in 2020 and 2021 had a higher hazard of death compared to patients diagnosed in 2019.

**Interpretation:**

After a continuous survival improvement for advanced melanoma patients between 2013 and 2019, outcomes of patients diagnosed in 2020 and 2021 seem poorer. This trend of decreased survival remained after correcting for known prognostic factors and previous neoadjuvant or adjuvant treatment, suggesting that it is explained by unmeasured factors, which—considering the timing—could be COVID-19-related.

**Funding:**

For the Dutch Melanoma Treatment Registry (DMTR), the Dutch Institute for Clinical Auditing foundation received a start-up grant from governmental organization The Netherlands Organization for Health Research and Development (ZonMW, project number 836002002). The DMTR is structurally funded by 10.13039/100002491Bristol-Myers Squibb, Merck Sharpe & Dohme, 10.13039/100004336Novartis, and Roche Pharma. Roche Pharma stopped funding in 2019, and 10.13039/100013226Pierre Fabre started funding the DMTR in 2019. For this work, no funding was granted.

**Research in context**

**Evidence before this study**

We searched PubMed using the terms “advanced melanoma”, “survival”, and “year” for studies published in English between January 1, 2015, and August 1, 2023. During this time period, the prognosis of advanced melanoma patients improved significantly. In the Netherlands, the median survival for these patients improved each year. However, the introduction of neoadjuvant and adjuvant treatment could influence advanced melanoma survival. Moreover, the COVID-19 pandemic disrupted healthcare systems around the world. As a consequence, diagnoses of melanoma were decreased and delayed. Advanced melanoma patients in the Netherlands have been shown to have worse disease characteristics during COVID-19. The consequences of neoadjuvant and adjuvant treatment and COVID-19 for the survival of advanced melanoma patients have not yet been investigated.

**Added value of this study**

Using a prospective, nationwide, population-based cancer registry, we evaluated the survival of advanced melanoma patients per year of diagnosis and described factors associated with the survival of these patients.

**Implications of all the available evidence**

Along with existing evidence, our findings help to put the survival of advanced melanoma patients in perspective and inform patients and clinicians on factors associated with survival. Further follow-up and investigation of this patient group is warranted to monitor the more long-term effects of our findings.

## Introduction

The prognosis of advanced (unresectable stage III and IV) melanoma patients has improved significantly over the last few years.^1^ The first immune checkpoint inhibitor (ICI) for the treatment of advanced melanoma was ipilimumab, a monoclonal antibody blocking CTLA-4 introduced in 2012.^2^ From 2012, BRAF inhibitors became available for patients with *BRAF*-mutant melanoma.^3^ Subsequently, anti-PD-1 antibodies were introduced in 2015, as well as the combination of BRAF inhibitors with MEK inhibitors and ipilimumab-nivolumab, both in 2016.^4^, ^5^, ^6^, ^7^ The treatment of advanced melanoma is still evolving with the introduction of new therapies, such as the lymphocyte-activation gene 3 (LAG-3) antibody and Tumor-Infiltrating Lymphocyte (TIL) therapy.^8^^,^^9^ In the Netherlands, the survival per year has improved since the introduction of checkpoint inhibition and targeted treatment for advanced melanoma.^10^ This improvement was also demonstrated for patients not represented in clinical trials.^11^ The question arises if the survival of real-world advanced melanoma patients will continue to improve. The introduction of neoadjuvant and adjuvant systemic therapy could influence the survival of advanced melanoma. In addition, our previous study demonstrated that advanced melanoma patients more often presented with worse performance status and brain metastases during the COVID-19 waves, which could result in poorer long-term outcomes.^12^ In this study, we investigate the survival of Dutch advanced melanoma patients per year of diagnosis and describe factors associated with the survival of these patients.

## Methods

Data were retrieved from the Dutch Melanoma Treatment Registry (DMTR). The DMTR prospectively registers data of all unresectable stage III and IV (i.e., advanced) melanoma patients in the Netherlands since 2013. For this study, the dataset cut-off date was August 9, 2023.

We included all patients with advanced melanoma (defined as unresectable stage III and IV melanoma), 18 years or older, from 2013 to 2021 and focused primarily on patients receiving systemic treatment. Patients were stratified by the year of diagnosis of their unresectable melanoma. Patients who developed unresectable disease after neoadjuvant or adjuvant treatment were included from their first treatment line for unresectable disease. Patients with uveal or mucosal melanoma were excluded.

### Patient and disease characteristics

The following patient and tumor characteristics at diagnosis were registered for all patients at the time of unresectable disease: age at diagnosis, sex, Eastern Cooperative Oncology Group Performance Status (ECOG PS), lactate dehydrogenase levels (LDH), location of primary melanoma, type of melanoma, Breslow thickness, ulceration, liver metastases, brain metastases, number of organ sites with metastases, American Joint Committee on Cancer (AJCC) staging system 8th edition,^13^ melanoma mutation status (*BRAF*, *NRAS*), synchronous or metachronous presentation (synchronous defined by the time between the diagnosis of the primary melanoma and the advanced melanoma of less than 90 days^14^), and type of systemic therapy.

### Statistical analysis

Baseline characteristics were analyzed using descriptive statistics. Pearson's chi-squared test was used to compare categorical variables, and the t-test for continuous variables. Median follow-up time was estimated from the diagnosis of advanced melanoma until the last moment of follow-up using the reversed Kaplan–Meier method.^15^ Median overall survival (OS) and melanoma-specific survival (MSS) were calculated using the Kaplan–Meier method. Starting point of the entire cohort was the date of diagnosis of advanced melanoma. The endpoint was death by any cause for OS, melanoma-related death for MSS, or the last moment of follow-up. Patients not reaching the endpoint were right-censored at the date of the last contact. A Cox proportional hazards model was used to estimate the association between different diagnosis years and OS in a multivariable analysis. Factors included in the model were age, gender, ECOG PS, LDH levels, liver metastasis, brain metastasis, number of organ sites with metastasis, prior adjuvant treatment, synchronous or metachronous diagnosis of primary and advanced melanoma, and year of diagnosis of advanced melanoma. These factors have been selected based on previous research using DMTR data.^16^, ^17^, ^18^ Patients with missing covariates were excluded from the multivariable Cox proportional hazards model.

Comparisons were considered statistically significant for two-sided P-values ≤ 0.05. Data handling and statistical analyses were performed using R (version 4.0.2),^19^ packages tidyverse,^20^ tableone,^21^ survival,^22^ and survminer.^23^

### Ethics statement

Research with DMTR data was approved by the medical ethical committee and was not deemed subject to the Medical Research Involving Human Subjects Act in compliance with Dutch regulations. Therefore, informed consent of the included patients was not required.

### Role of the funding source

For this work, no funding was received. O.J. van Not and M Bloem had access to the dataset. All coauthors approved the decision to submit the article for publication.

## Results

**Fig. 1:**
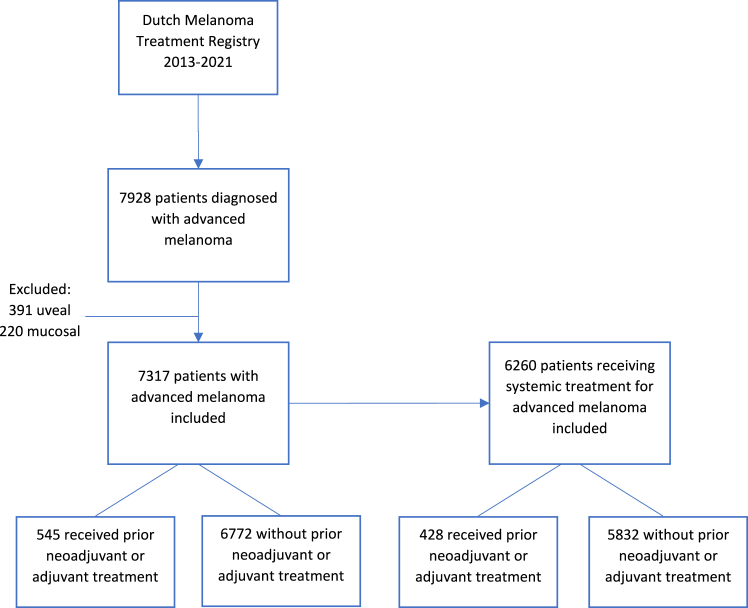
Flowchart of included patients.

From 2013 to 2021, 7928 patients with advanced melanoma were registered in the DMTR regardless of receiving systemic treatment. We excluded 391 patients with uveal melanoma and 220 patients with mucosal melanoma from this cohort, which resulted in 7317 included patients. The percentage of advanced melanoma patients receiving systemic treatment was 74% in 2013, 86% in 2020, and was 83% in 2021 [Supplementary Table S1]. During these years, 6596 patients received systemic treatment for advanced melanoma. We excluded 168 patients with uveal melanoma and 168 with mucosal melanoma. Of the 6260 included patients, 428 received neoadjuvant or adjuvant treatment prior to receiving systemic treatment for advanced melanoma [Fig. 1].

Median follow-up was 50.9 months (95% CI 49.4–52.5), ranging from a median follow-up of 106.0 months (95% CI 103.1–109.1) for patients diagnosed in 2013 to 14.1 months (95% CI 13.4–14.7) for patients diagnosed in 2021. In patients developing advanced-stage melanoma after prior neoadjuvant or adjuvant treatment, median follow-up from the diagnosis of unresectable stage melanoma was 23.3 months (95% CI 21.4–25.5), ranging from 40.7 months (95% CI 36.4–41.9) for the 2019 cohort to 13.6 months (95% CI 11.3–15.2) for the 2021 cohort.

### Patient and tumor characteristics

Baseline characteristics of systemically treated patients, stratified by year of diagnoses of advanced disease, are shown in [Table 1]. The median age of patients diagnosed with advanced melanoma gradually increased from 60 years in 2013 to 66 years in 2021, even though melanoma also increasingly affects younger people. The number of patients with an ECOG PS ≥ 2 increased. In 2013, only 10% of patients had an ECOG PS of ≥2 vs. 14% in 2021. More patients diagnosed with brain metastasis were treated, starting from 24% in 2013, while 28% had brain metastases in 2021. LDH levels did not show a trend across the years. However, over time and especially in 2020 and 2021, more patients presented with synchronous (<90 days) metastatic disease (10% in 2013, 29% in 2020, and 34% in 2021) [Supplementary Fig. S1].

**Table 1:** Patient characteristics.

	2013	2014	2015	2016	2017	2018	2019	2020	2021
n	595	574	679	675	702	771	790	737	737
Age									
<70 years	462 (77.6)	428 (74.6)	488 (71.9)	457 (67.7)	464 (66.1)	461 (59.8)	464 (58.7)	415 (56.3)	432 (58.6)
≥70 years	133 (22.4)	146 (25.4)	191 (28.1)	218 (32.3)	238 (33.9)	310 (40.2)	326 (41.3)	322 (43.7)	305 (41.4)
Median age [IQR]	60.0 [49.0, 69.0]	62.00 [53.0, 70.0]	62.0 [52.0, 71.0]	64.0 [54.0, 72.0]	64.0 [54.0, 72.0]	65.0 [55.0, 74.0]	66.0 [56.0, 75.0]	67.0 [56.0, 75.0]	66.0 [55.0, 75.0]
Sex									
Male	337 (56.6)	330 (57.5)	423 (62.3)	402 (59.6)	412 (58.7)	473 (61.3)	486 (61.5)	447 (60.7)	442 (60.0)
Female	258 (43.4)	244 (42.5)	256 (37.7)	273 (40.4)	290 (41.3)	298 (38.7)	304 (38.5)	290 (39.3)	295 (40.0)
ECOG PS									
0	316 (53.1)	297 (51.7)	367 (54.1)	340 (50.4)	324 (46.2)	348 (45.1)	335 (42.4)	320 (43.4)	338 (45.9)
1	172 (28.9)	168 (29.3)	195 (28.7)	214 (31.7)	221 (31.5)	273 (35.4)	302 (38.2)	258 (35.0)	243 (33.0)
≥2	60 (10.1)	54 (9.4)	71 (10.5)	74 (11.0)	96 (13.7)	83 (10.8)	95 (12.0)	105 (14.2)	101 (13.7)
Unknown	47 (7.9)	55 (9.6)	46 (6.8)	47 (7.0)	61 (8.7)	67 (8.7)	58 (7.3)	54 (7.3)	55 (7.5)
Melanoma location									
Primary unknown	62 (10.4)	93 (16.2)	93 (13.7)	97 (14.4)	102 (14.5)	107 (13.9)	120 (15.2)	110 (14.9)	114 (15.5)
Head-Neck	65 (10.9)	67 (11.7)	88 (13.0)	89 (13.2)	97 (13.8)	108 (14.0)	116 (14.7)	89 (12.1)	101 (13.7)
Trunk	254 (42.7)	219 (38.2)	282 (41.5)	241 (35.7)	270 (38.5)	328 (42.5)	320 (40.5)	301 (40.8)	312 (42.3)
Extremities	193 (32.4)	168 (29.3)	191 (28.1)	228 (33.8)	212 (30.2)	211 (27.4)	204 (25.8)	210 (28.5)	176 (23.9)
Acral	15 (2.5)	22 (3.8)	19 (2.8)	18 (2.7)	20 (2.8)	11 (1.4)	18 (2.3)	18 (2.4)	20 (2.7)
Melanoma type									
Superficial spreading	269 (45.2)	219 (38.2)	312 (45.9)	307 (45.5)	286 (40.7)	311 (40.3)	324 (41.0)	297 (40.3)	308 (41.8)
Nodular	133 (22.4)	143 (24.9)	146 (21.5)	138 (20.4)	150 (21.4)	140 (18.2)	119 (15.1)	146 (19.8)	121 (16.4)
Acral lentiginous	12 (2.0)	11 (1.9)	16 (2.4)	15 (2.2)	19 (2.7)	8 (1.0)	21 (2.7)	12 (1.6)	12 (1.6)
Lentigo maligna	5 (0.8)	8 (1.4)	9 (1.3)	9 (1.3)	14 (2.0)	19 (2.5)	7 (0.9)	13 (1.8)	17 (2.3)
Desmoplastic	2 (0.3)	1 (0.2)	6 (0.9)	7 (1.0)	3 (0.4)	1 (0.1)	3 (0.4)	4 (0.5)	2 (0.3)
Other	41 (6.9)	31 (5.4)	23 (3.4)	19 (2.8)	26 (3.7)	14 (1.8)	21 (2.7)	6 (0.8)	7 (0.9)
Unknown	133 (22.4)	161 (28.0)	167 (24.6)	180 (26.7)	204 (29.1)	278 (36.0)	295 (37.3)	259 (35.1)	270 (36.6)
Median Breslow thickness [IQR]	2.1 [1.3, 4.0]	2.3 [1.3, 4.0]	2.2 [1.3, 4.0]	2.5 [1.4, 4.2]	2.5 [1.4, 4.1]	2.4 [1.4, 4.1]	2.4 [1.4, 4.7]	2.5 [1.4, 4.4]	2.5 [1.4, 4.1]
Liver metastases									
No	387 (65.0)	392 (68.3)	462 (68.0)	473 (70.1)	481 (68.5)	553 (71.7)	561 (71.0)	551 (74.8)	533 (72.3)
Yes	199 (33.4)	174 (30.3)	208 (30.6)	194 (28.7)	212 (30.2)	210 (27.2)	223 (28.2)	180 (24.4)	188 (25.5)
Unknown	9 (1.5)	8 (1.4)	9 (1.3)	8 (1.2)	9 (1.3)	8 (1.0)	6 (0.8)	6 (0.8)	16 (2.2)
Brain metastases									
No	450 (75.6)	421 (73.3)	498 (73.3)	502 (74.4)	492 (70.1)	539 (69.9)	574 (72.7)	532 (72.2)	523 (71.0)
Yes, asymptomatic	63 (10.6)	62 (10.8)	71 (10.5)	66 (9.8)	86 (12.3)	119 (15.4)	115 (14.6)	115 (15.6)	92 (12.5)
Yes, symptomatic	76 (12.8)	87 (15.2)	108 (15.9)	106 (15.7)	124 (17.7)	109 (14.1)	100 (12.7)	90 (12.2)	114 (15.5)
Unknown	6 (1.0)	4 (0.7)	2 (0.3)	1 (0.1)	0 (0.0)	4 (0.5)	1 (0.1)	0 (0.0)	8 (1.1)
AJCC stage (8th edition)									
IIIc unresectable	20 (3.4)	19 (3.3)	39 (5.7)	43 (6.4)	55 (7.8)	72 (9.3)	96 (12.2)	149 (20.2)	155 (21.0)
IV-M1a	60 (10.1)	54 (9.4)	65 (9.6)	68 (10.1)	38 (5.4)	67 (8.7)	55 (7.0)	42 (5.7)	40 (5.4)
IV-M1b	64 (10.8)	65 (11.3)	77 (11.3)	81 (12.0)	88 (12.5)	88 (11.4)	91 (11.5)	89 (12.1)	66 (9.0)
IV-M1c	306 (51.4)	283 (49.3)	317 (46.7)	310 (45.9)	311 (44.3)	312 (40.5)	332 (42.0)	252 (34.2)	262 (35.5)
IV-M1d	139 (23.4)	149 (26.0)	179 (26.4)	172 (25.5)	210 (29.9)	228 (29.6)	215 (27.2)	205 (27.8)	206 (28.0)
Unknown	6 (1.0)	4 (0.7)	2 (0.3)	1 (0.1)	0 (0.0)	4 (0.5)	1 (0.1)	0 (0.0)	8 (1.1)
LDH levels									
Not determined	20 (3.4)	20 (3.5)	20 (2.9)	13 (1.8)	15 (2.1)	19 (2.4)	20 (2.5)	40 (5.4)	30 (4.1)
Normal	397 (66.7)	380 (66.2)	408 (60.1)	391 (57.9)	418 (59.5)	455 (59.0)	497 (62.9)	445 (60.4)	467 (63.4)
250–500	95 (16.0)	105 (18.3)	156 (23.0)	200 (29.6)	191 (27.2)	181 (23.5)	171 (21.6)	169 (22.9)	164 (22.3)
>500	83 (13.9)	69 (12.0)	95 (14.0)	71 (10.5)	78 (11.1)	116 (15.0)	102 (12.9)	83 (11.3)	76 (10.3)
Organ sites									
<3	288 (48.4)	260 (45.3)	352 (51.8)	335 (49.6)	353 (50.3)	393 (51.0)	428 (54.2)	450 (61.1)	434 (58.9)
≥3	303 (50.9)	310 (54.0)	325 (47.9)	337 (49.9)	347 (49.4)	370 (48.0)	359 (45.4)	284 (38.5)	296 (40.2)
Unknown	4 (0.7)	4 (0.7)	2 (0.3)	3 (0.4)	2 (0.3)	8 (1.0)	3 (0.4)	3 (0.4)	7 (0.9)
*BRAF* mutation									
Yes	403 (67.7)	344 (59.9)	422 (62.2)	390 (57.8)	428 (61.0)	455 (59.0)	433 (54.8)	402 (54.5)	422 (57.3)
No/not determined	192 (32.3)	230 (40.1)	257 (37.8)	285 (42.2)	274 (39.0)	316 (41.0)	357 (45.2)	335 (45.5)	315 (42.7)
*NRAS* mutation									
Yes	71 (11.9)	110 (19.2)	116 (17.1)	159 (23.6)	137 (19.5)	149 (19.3)	171 (21.6)	150 (20.4)	144 (19.5)
No/not determined	524 (88.1)	464 (80.8)	563 (82.9)	516 (76.4)	565 (80.5)	622 (80.7)	619 (78.4)	587 (79.6)	593 (80.5)
Time between primary and advanced melanoma (days)									
≤90	60 (10.1)	97 (16.9)	122 (18.0)	123 (18.2)	113 (16.1)	140 (18.2)	191 (24.2)	215 (29.2)	247 (33.5)
>90	534 (89.7)	477 (83.1)	557 (82.0)	552 (81.8)	589 (83.9)	631 (81.8)	599 (75.8)	522 (70.8)	490 (66.5)
Type of systemic therapy in first systemic treatment line for irresectable stage melanoma									
Chemotherapy	83 (13.9)	61 (10.6)	11 (1.6)	3 (0.4)	1 (0.1)	0 (0.0)	3 (0.4)	1 (0.1)	0 (0.0)
BRAF inhibitor	277 (46.6)	198 (34.5)	184 (27.1)	46 (6.8)	9 (1.3)	1 (0.1)	6 (0.8)	2 (0.3)	4 (0.5)
BRAF/MEK inhibitors	52 (8.7)	25 (4.4)	60 (8.8)	175 (25.9)	223 (31.8)	211 (27.4)	213 (27.0)	199 (27.0)	216 (29.3)
Ipilimumab	105 (17.6)	229 (39.9)	194 (28.6)	27 (4.0)	5 (0.7)	1 (0.1)	10 (1.3)	30 (4.1)	22 (3.0)
Anti-PD-1 antibody	7 (1.2)	16 (2.8)	170 (25.0)	343 (50.8)	322 (45.9)	339 (44.0)	314 (39.7)	244 (33.1)	224 (30.4)
Ipilimumab-nivolumab	1 (0.2)	0 (0.0)	7 (1.0)	48 (7.1)	103 (14.7)	157 (20.4)	210 (26.6)	212 (28.8)	226 (30.7)
T-VEC	0 (0.0)	0 (0.0)	2 (0.3)	0 (0.0)	14 (2.0)	16 (2.1)	25 (3.2)	34 (4.6)	28 (3.8)
Other	70 (11.8)	45 (7.8)	51 (7.5)	33 (4.8)	25 (3.6)	46 (6.0)	9 (1.1)	15 (2.0)	17 (2.3)

Comparison of baseline characteristics of all systemically treated patients with advanced melanoma stratified by year of diagnosis.

ECOG PS: Eastern Cooperative Oncology Group Performance Status, AJCC: American Joint Committee on Cancer, LDH: lactate dehydrogenase.

Naturally, the type of systemic therapies also changed over the years. In earlier years, BRAF inhibitors, ipilimumab monotherapy, and chemotherapy were most frequently given as a treatment. In more recent years, this has shifted to BRAF/MEK inhibitors, anti-PD-1 antibodies, and ipilimumab-nivolumab. The duration of anti-PD-1 treatment and BRAF/MEK decreased over time, while treatment duration for other treatments remained relatively stable over time [Supplementary Table S2].

Baseline characteristics of patients developing advanced melanoma after prior neoadjuvant or adjuvant treatment did not show significant changes over the years [Supplementary Table S3]. Baseline characteristics of advanced melanoma patients without prior neoadjuvant or adjuvant treatment can be found in [Supplementary Table S4].

### Overall and melanoma-specific survival

**Fig. 2:**
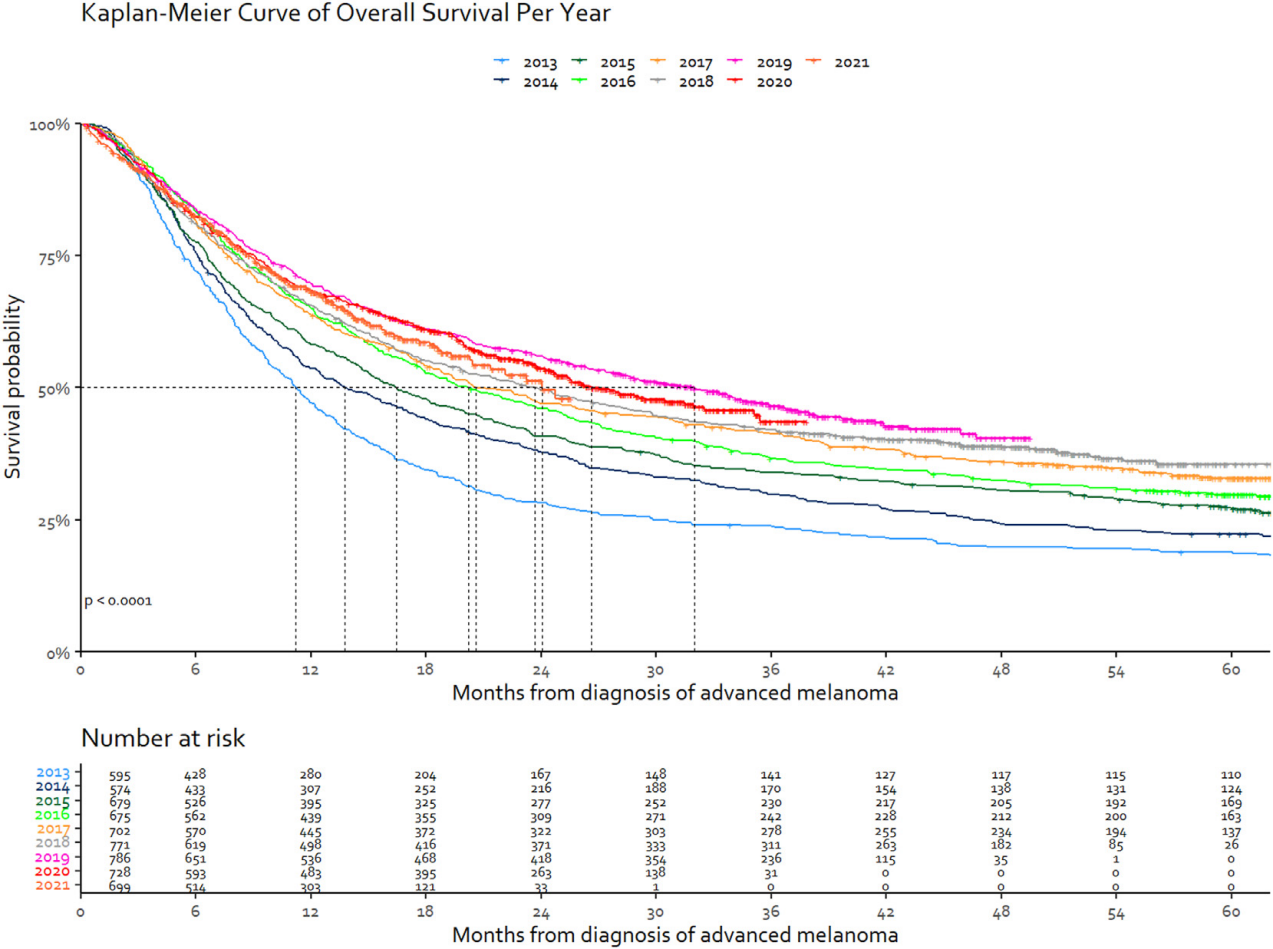
Kaplan–Meier estimate∗ of the overall survival of systemically treated patients with advanced melanoma stratified by diagnosis year. ∗Kaplan–Meier curve is abrogated when less than 20 patients are left at risk.

Median OS of advanced melanoma patients who received systemic treatment was 11.2 months (95% CI 10.0–12.4) for the 2013 cohort. The median OS gradually increased, reaching a maximum of 32.0 months (95% CI 26.6–36.7) in patients diagnosed in 2019 [Fig. 2]. Patients diagnosed in 2020 and 2021 showed a non-significant (P = 0.27) lower survival compared to 2019, with a median OS of 26.6 months (95% CI 23.9–35.1) and 24.0 months (95% CI 20.4-NR), respectively. Although this was not a significant difference, it did clearly break the upward trend in survival that we observed in the years before. The same trend was observed in MSS: median MSS was 14.0 months (95% CI 12.2–16.2) for the 2013 cohort and increased to 41.9 months (95% CI 35.6-NR) for the 2019 cohort. The median MSS for the 2020 cohort was not reached and for the 2021 cohort the median MSS was 28.3 months (95% CI 28.3-NR), but the Kaplan–Meier curves of 2020 and 2021 show the same trend as the OS curves [Supplementary Fig. S2]. Similar differences were observed for all advanced melanoma patients, regardless of systemic treatment [Supplementary Fig. S3].

### Neoadjuvant and adjuvant treatment

**Fig. 3:**
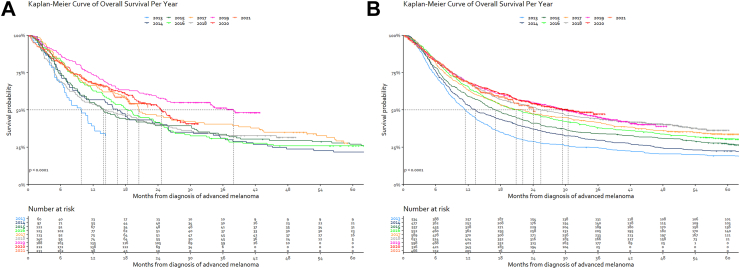
A—Kaplan–Meier estimate∗ of the overall survival of systemically treated patients with a synchronously diagnosed (<90 days after primary melanoma) advanced melanoma stratified by diagnosis year. ∗Kaplan–Meier curve is abrogated when less than 20 patients are left at risk. B—Kaplan–Meier estimate∗ of the overall survival of systemically treated patients with a metachronous diagnosed (≥90 days after primary melanoma) advanced melanoma stratified by diagnosis year. ∗Kaplan–Meier curve is abrogated when less than 20 patients are left at risk.

Median OS from diagnosis of advanced disease did not differ significantly over the years when focusing only on advanced melanoma patients who received prior (neo) adjuvant treatment (p = 0.14) [Supplementary Fig. S4]. Similar changes in OS over time were observed when we excluded patients who received neoadjuvant or adjuvant treatment before developing unresectable disease. Patients with synchronous metastases had a shorter median OS than those with metachronous metastases (17.8 months vs. 20.6 months; p = 0.0039; HR 0.86, 95% CI 0.79–0.93) [Supplementary Fig. S5]. The same trend in median OS was observed in patients with synchronous diagnosis only, similar to the entire cohort. The log-rank P-value for comparing 2019, 2020, and 2021 was 0.059 [Fig. 3A]. The differences in OS over the different years of diagnosis were not as apparent in patients with metachronous disease [Fig. 3B].

### Multivariable cox analysis

**Fig. 4:**
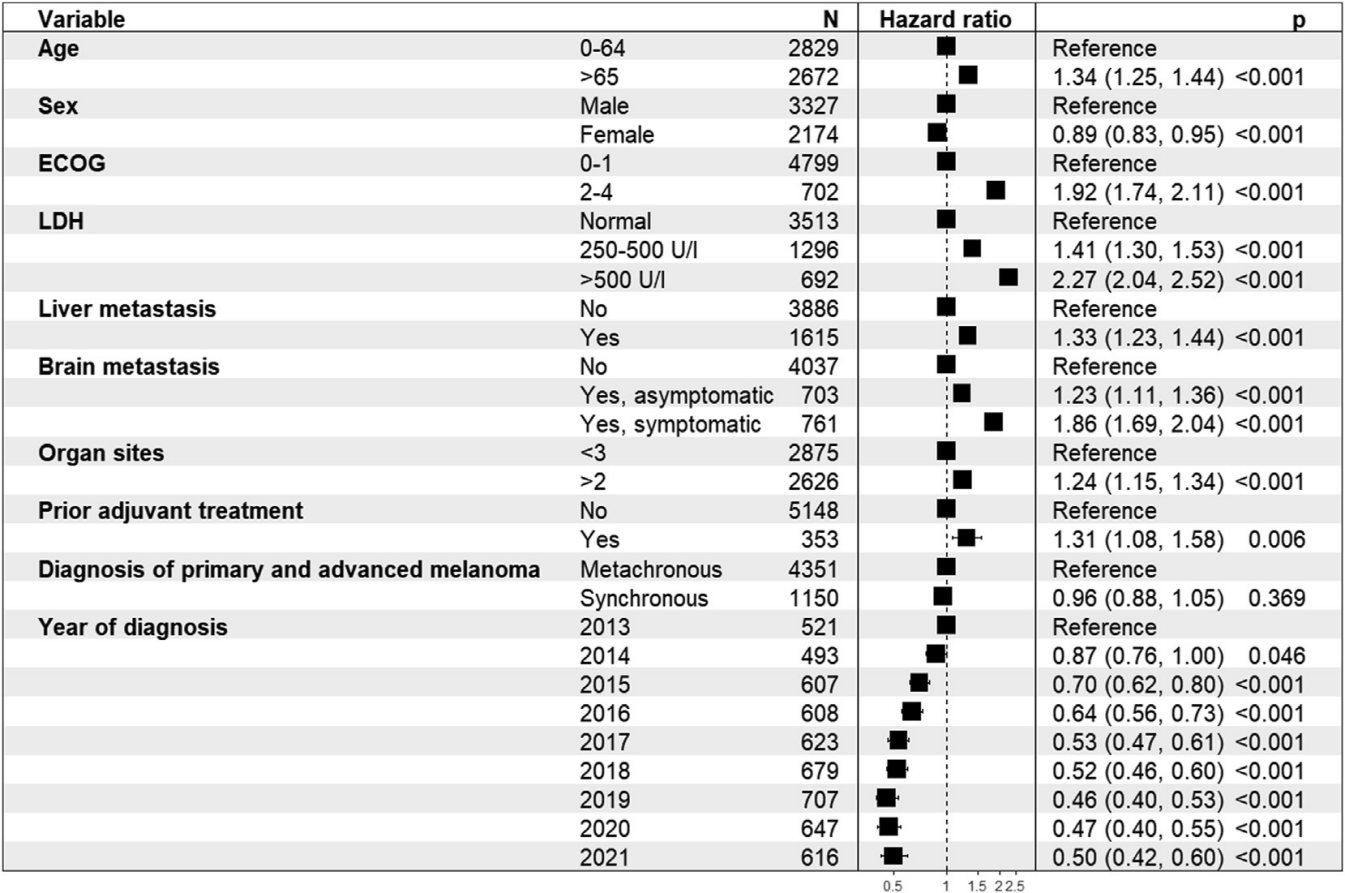
Multivariable Cox proportional hazard model of the overall survival of systemically treated patients with advanced melanoma.

The multivariable Cox analysis of OS showed a significantly lower hazard of death for the years of diagnosis after 2013. However, patients diagnosed in 2020 and 2021 showed a non-significant trend towards a higher hazard of death than patients diagnosed in 2019 [Fig. 4]. The HR_adj_ for 2020 and 2021 compared to 2019 were 1.05 (95% CI 0.90–1.23) and 1.12 (95% CI 0.94–1.34), respectively. Progressing to irresectable stage melanoma after prior adjuvant treatment was also associated with a higher risk of death (HR_adj_ 1.31; 95% CI 1.08–1.58; p = 0.006). In multivariable analysis, having a synchronous or metachronous diagnosis was no longer significantly associated with a higher risk of death.

## Discussion

The Dutch advanced melanoma patient population has demonstrated a gradual increase in survival rates over the past decade. However, as of 2020, this upward trend has stopped. After the exclusion of neoadjuvant and adjuvant-treated patients or when focusing on patients with synchronous metastases only, we observed similar trends. Moreover, this trend remains after adjusting for prognostic factors, previous neoadjuvant and adjuvant treatment or synchronous presentation in multivariable analysis. This suggests that the decrease in survival after 2019 is not, or at least not completely, explained by the introduction of neoadjuvant and adjuvant treatment. With more patients developing metastases after neoadjuvant and adjuvant treatment, it is important to investigate how neoadjuvant and adjuvant patients progressing to unresectable melanoma respond to systemic melanoma treatment. Our multivariable Cox analysis showed an increased hazard of death for patients with prior neoadjuvant and adjuvant treatment. We are currently conducting a study to further evaluate the outcomes of this patient group. The decrease in survival is not caused by doctors treating patients with worse patient and tumor characteristics because the same survival trends are observed in all advanced melanoma patients, regardless of systemic treatment. Even though the treatment duration of anti-PD-1 has been shorter in more recent years, this does not seem to explain our findings since there has been no sharp decrease in treatment duration after 2019. Moreover, there were no substantial changes in other baseline characteristics, such as age or ECOG PS, after 2019, which could explain our findings. In our data, we saw a gradual decrease in patients treated with targeted therapy and a gradual increase in first-line treatment with ipilimumab-nivolumab. Since the latter has been proven to be a superior first-line strategy in clinical trials.^24^^,^^25^ This might partly explain the gradual increase in survival until 2019 but cannot explain the decrease after 2019. In our cohort, patients with a synchronous primary and advanced melanoma diagnosis had shorter median OS than those with a metachronous diagnosis. We noticed an increase in the number and percentage of patients presenting with synchronous diagnoses over the years. Especially in 2020 and 2021, more patients presented with synchronous diagnoses. The difference in survival is not as evident in the metachronous diagnosed patients. However, when adjusting for synchronous/metachronous presentation in the multivariable analysis, the pattern of increased risk of death in 2021 and 2022 remained. The results of the Cox analysis did not show a significant increase in the risk of death associated with synchronous diagnosis, possibly because patients with synchronous diagnosis had poorer baseline characteristics than those with metachronous diagnosis (data not shown). The increasing proportion of patients with a synchronous diagnosis might be (partly) caused by the COVID-19 pandemic. The pandemic caused a delay in cancer diagnoses, leading to a larger proportion of patients presenting with metastatic disease.^26^^,^^27^

In a previous study that investigated the effects of COVID-19 on advanced melanoma care in the Netherlands, we observed fewer diagnoses of advanced melanoma during the COVID pandemic and an increase in patients presenting with brain metastases and poorer performance status at diagnosis of advanced disease.^12^ We also reported that during COVID-19, the time between diagnosis of advanced melanoma and start of systemic treatment was longer in regions heavily affected by COVID-19 and that anti-PD-1 courses were postponed,^12^ which might also affect survival. The survival of advanced melanoma patients diagnosed after the pandemic in 2022 and 2023 will teach us more about the role that COVID-19 plays in the findings of this study.

Our study has several limitations. First, follow-up for the 2020 and 2021 cohorts is shorter than prior cohorts, which can affect outcomes, especially in the long term. Second, comparing multiple diagnosis years could t heoretically lead to type I errors due to multiple comparisons. The variables used in multivariable Cox proportional hazards model were selected based on clinical experience^16^, ^17^, ^18^ and the inclusion of these variables did not depend on univariable significant association. Third, the results of our study must be generalized with care. Since various countries have adopted differently to the pandemic, the diagnosis and therapy of melanoma were impacted differently across the world. Fourth, since data from the DMTR are observational data, this may lead to bias by indication due to the choice of therapy by the treating physician. Nonetheless, the data of the DMTR have been shown to be of high quality, with 93% of patients having no missing values on baseline characteristics.^28^^,^^29^ Data are registered by independent data managers. The treating physician validates the data to further ensure quality. The online registry in which data are registered also warns for inconsistent data or missing values. Moreover, the DMTR covers all advanced melanoma patients in the Netherlands. This makes selection bias unlikely to explain the differences found.

Concluding, after a consequent improvement in survival of advanced melanoma patients between 2013 and 2019, this upward trend has stopped for patients diagnosed in 2020 and 2021. This negative trend in the last two years remained after correcting for known prognostic factors and previous neoadjuvant and adjuvant treatment, suggesting that it is explained by unmeasured residual factors, which—considering the timing—might be COVID-related. The extended follow-up of this patient group and subsequent treatment years are essential to further put these findings into perspective. Our data stress the importance of ongoing innovation in melanoma treatment to improve the prognosis of advanced melanoma patients. This study gives insight into the survival of patients with advanced melanoma over the years and can be of value to inform patients and clinicians about survival-associated factors.

## Contributors

Van Not: Conceptualization, Data curation, Formal analysis, Methodology, Investigation, Project administration, Writing–Original Draft, Writing–Review & Editing.

Van den Eertwegh: Investigation, Project administration, Writing–Review & Editing.

Haanen: Investigation, Project administration, Writing–Review & Editing.

Blank: Investigation, Project administration, Writing–Review & Editing.

Aarts: Investigation, Project administration, Writing–Review & Editing.

Van Breeschoten: Formal Analysis, Investigation, Project administration, Writing–Review & Editing.

Van den Berkmortel: Investigation, Project administration, Writing–Review & Editing.

De Groot: Investigation, Project administration, Writing–Review & Editing.

Hospers: Investigation, Project administration, Writing–Review & Editing.

Ismail: Investigation, Project administration, Writing–Review & Editing.

Kapiteijn: Investigation, Project administration, Writing–Review & Editing.

Bloem: Investigation, Project administration, Writing–Review & Editing.

De Meza: Investigation, Project administration, Writing–Review & Editing.

Piersma: Investigation, Project administration, Writing–Review & Editing.

Van Rijn: Investigation, Project administration, Writing–Review & Editing.

Stevense-den Boer: Investigation, Project administration, Writing–Review & Editing.

Van der Veldt: Investigation, Project administration, Writing–Review & Editing.

Vreugdenhil: Investigation, Project administration, Writing–Review & Editing.

Boers-Sonderen: Investigation, Project administration, Writing–Review & Editing.

Blokx: Conceptualization, Methodology, Investigation, Project administration, Writing–Review & Editing, Supervision.

Wouters: Conceptualization, Methodology, Investigation, Project administration, Writing–Review & Editing, Supervision.

Suijkerbuijk: Conceptualization, Methodology, Investigation, Project administration, Writing–Review & Editing, Supervision.

## Data sharing statement

Data from the Dutch Melanoma Treatment Registry are not publicly available but are available upon reasonable request.

## Declaration of interests

AvdE has advisory relationships with Amgen, Bristol Myers Squibb, Roche, Novartis, MSD, Pierre Fabre, Sanofi, Pfizer, Ipsen, Merck and has received research study grants not related to this paper from Sanofi, Bristol Myers Squibb, Idera and TEVA and has received travel expenses from MSD Oncology, Roche, Pfizer, Pierre Fabre, and Sanofi.

JH has advisory relationships with AstraZeneca, Achilles Therapeutics, BioNTech, BMS, CureVac, Iovance Bio, Eisai, Instil Bio, Imcyse, MSD, Neogene Therapeutics, Novartis, Roche, Sanofi, Sastra Cell Therapy, TRV, and T-Knife. And has received research grants not related to this paper from Asher Bio, Amgen, Bristol Myers Squibb, BioNTech, Novartis, and Sastra Cell Therapy. All grants were paid to the institutions.

MA has advisory board/consultancy honoraria from Amgen, Bristol Myers Squibb, Novartis, MSD-Merck, Merck-Pfizer, Pierre Fabre, Sanofi, Astellas, Bayer. She received travel expenses from Astella, BMS, Janssen, Pfizer, and Sanofi and a research grant from Merck-Pfizer. Not related to current work and paid to institute.

GH consultancy/advisory relationships with Amgen, Bristol Myers Squibb, Roche, MSD, Pfizer, Novartis, Sanofi, Pierre Fabre and has received research grants not related to this paper from Bristol Myers Squibb and Seerave. All payments were paid to the institution.

RI has no declarations of interest for this research, but is employed at MSD since January 2022.

DP has advisory relationships/consultancy honororia with Pierre Fabre and Novartis and received travel expenses from Pierre Fabre.

AvdV has consultancy relationships with Bristol Myers Squibb, MSD, Roche, Novartis, Pierre Fabre, Pfizer, Sanofi, Ipsen, Eisai all paid to the institute.

KS has consulting/advisory relationship with Abbvie, Pierre Fabre, and Sairopa. She received honoraria received from Novartis, Roche, Merck Sharp and Dome and research funding not related to this paper from Genmab, TigaTx, Bristol Myers Squibb, and Philips. All paid to institution.

All remaining authors have declared no conflicts of interest.

## References

[1] Ugurel S., Röhmel J., Ascierto P.A. (2016). Survival of patients with advanced metastatic melanoma: the impact of novel therapies. Eur J Cancer.

[2] Wolchok J.D., Hodi F.S., Weber J.S. (2013). Development of ipilimumab: a novel immunotherapeutic approach for the treatment of advanced melanoma. Ann N Y Acad Sci.

[3] Hauschild A., Grob J.J., Demidov L.V. (2012). Dabrafenib in BRAF-mutated metastatic melanoma: a multicentre, open-label, phase 3 randomised controlled trial. Lancet.

[4] Robert C., Schachter J., Long G.V. (2015). Pembrolizumab versus ipilimumab in advanced melanoma. N Engl J Med.

[5] Dummer R., Ascierto P.A., Gogas H.J. (2018). Encorafenib plus binimetinib versus vemurafenib or encorafenib in patients with BRAF-mutant melanoma (COLUMBUS): a multicentre, open-label, randomised phase 3 trial. Lancet Oncol.

[6] Wolchok J.D., Kluger H., Callahan M.K. (2013). Nivolumab plus ipilimumab in advanced melanoma. N Engl J Med.

[7] Larkin J., Chiarion-Sileni V., Gonzalez R. (2019). Five-year survival with combined nivolumab and ipilimumab in advanced melanoma. N Engl J Med.

[8] Tawbi H.A., Schadendorf D., Lipson E.J. (2022). Relatlimab and nivolumab versus nivolumab in untreated advanced melanoma. N Engl J Med.

[9] Rohaan M.W., Borch T.H., van den Berg J.H. (2022). Tumor-infiltrating lymphocyte therapy or ipilimumab in advanced melanoma. N Engl J Med.

[10] van Zeijl M.C.T., de Wreede L.C., van den Eertwegh A.J.M. (2021). Survival outcomes of patients with advanced melanoma from 2013 to 2017: results of a nationwide population-based registry. Eur J Cancer.

[11] van Zeijl M.C.T., Ismail R.K., de Wreede L.C. (2020). Real-world outcomes of advanced melanoma patients not represented in phase III trials. Int J Cancer.

[12] van Not O.J., van Breeschoten J., van den Eertwegh A.J.M. (2022). The unfavorable effects of COVID-19 on Dutch advanced melanoma care. Int J Cancer.

[13] Gershenwald J.E., Scolyer R.A., Hess K.R. (2017). Melanoma staging: evidence-based changes in the American Joint Committee on Cancer eighth edition cancer staging manual. CA Cancer J Clin.

[14] Ng W.W.C., Cheung Y.S., Wong J., Lee K.F., Lai P.B.S. (2009). A preliminary analysis of combined liver resection with new chemotherapy for synchronous and metachronous colorectal liver metastasis. Asian J Surg.

[15] Schemper M., Smith T.L. (1996). A note on quantifying follow-up in studies of failure time. Control Clin Trials.

[16] van Breeschoten J., Wouters M.W.J.M., Hilarius D.L. (2021). First-line BRAF/MEK inhibitors versus anti-PD-1 monotherapy in BRAFV600-mutant advanced melanoma patients: a propensity-matched survival analysis. Br J Cancer.

[17] Ismail R.K., Sikkes N.O., Wouters M.W.J.M. (2021). Postapproval trials versus patient registries: comparability of advanced melanoma patients with brain metastases. Melanoma Res.

[18] Van Zeijl M.C.T., Haanen J.B.A.G., Wouters M.W.J.M. (2020). Real-world outcomes of first-line anti-PD-1 therapy for advanced melanoma: a nationwide population-based study. J Immunother.

[19] R Core Team (2017).

[20] Wickham H., Averick M., Bryan J. (2019). Welcome to the tidyverse. J Open Source Softw.

[21] Yoshida K., Bartel A. (2020).

[22] Therneau Terry M., Grambsch P.M. (2000).

[23] Kassambra Alboukadel, Kosinski Marcin, Biecek P. (2020).

[24] Ascierto P.A., Mandalà M., Ferrucci P.F. (2023). Sequencing of ipilimumab plus nivolumab and encorafenib plus binimetinib for untreated BRAF -mutated metastatic melanoma (SECOMBIT): a randomized, three-arm, open-label phase II trial. J Clin Oncol.

[25] Atkins M.B., Lee S.J., Chmielowski B. (2023). Combination dabrafenib and trametinib versus combination nivolumab and ipilimumab for patients with advanced BRAF -mutant melanoma: the DREAMseq trial - ECOG-ACRIN EA6134. J Clin Oncol.

[26] Riera R., Bagattini Â.M., Pacheco R.L., Pachito D.V., Roitberg F., Ilbawi A. (2021). Delays and disruptions in cancer Health care due to COVID-19 pandemic: systematic review. JCO Glob Oncol.

[27] Seretis K., Bounas N., Gaitanis G. (2022).

[28] Jochems A., Schouwenburg M.G., Leeneman B. (2017). Dutch Melanoma Treatment Registry: quality assurance in the care of patients with metastatic melanoma in The Netherlands. Eur J Cancer.

[29] de Meza M.M., Ismail R.K., Rauwerdink D. (2021). Adjuvant treatment for melanoma in clinical practice – trial versus reality. Eur J Cancer.

