# Supplementary material for: Improving survival in advanced melanoma patients: a trend analysis from 2013 to 2021
Source: eClinicalMedicine. 2024 Feb 12;69:102485. doi: 10.1016/j.eclinm.2024.102485 (PMC10874714; doi:10.1016/j.eclinm.2024.102485)
Supplement: Supplementary Figs. S1–S5 and Tables S1–S4 [file mmc1.docx]

# **SUPPLEMENTARY FILES**

## **Supplementary Table 1 – Patient Characteristics** Comparison of baseline characteristics of all advanced melanoma patients, regardless of receiving systemic treatment.

|  |  | 2013 | 2014 | 2015 | 2016 | 2017 | 2018 | 2019 | 2020 | 2021 |
| --- | --- | --- | --- | --- | --- | --- | --- | --- | --- | --- |
| N |  | 711 | 748 | 830 | 811 | 790 | 871 | 874 | 817 | 865 |
| Systemic treatment | Yes | 529 (74.4) | 491 (65.6) | 595 (71.7) | 611 (75.3) | 630 (79.7) | 704 (80.8) | 724 (82.8) | 702 (85.9) | 721 (83.4) |
| Age | <70 years | 527 (74.1) | 501 (67.0) | 553 (66.6) | 514 (63.4) | 498 (63.0) | 493 (56.6) | 484 (55.4) | 451 (55.2) | 469 (54.2) |
|  | ≥70 years | 184 (25.9) | 247 (33.0) | 277 (33.4) | 297 (36.6) | 292 (37.0) | 378 (43.4) | 390 (44.6) | 366 (44.8) | 396 (45.8) |
| Median age [IQR] |  | 61.0 [50.0, 70.0] | 64.0 [55.0, 72.0] | 64.0 [53.0, 72.0] | 65.0 [55.0, 73.0] | 65.0 [55.0, 73.0] | 66.0 [56.0, 75.0] | 67.0 [56.0, 76.0] | 67.0 [57.0, 75.0] | 68.0 [56.0, 76.0] |
| Sex | Male | 396 (55.7) | 442 (59.1) | 513 (61.8) | 484 (59.7) | 460 (58.2) | 530 (60.8) | 531 (60.8) | 499 (61.1) | 509 (58.8) |
|  | Female | 315 (44.3) | 306 (40.9) | 317 (38.2) | 327 (40.3) | 330 (41.8) | 341 (39.2) | 343 (39.2) | 318 (38.9) | 356 (41.1) |
| ECOG PS | 0 | 343 (48.2) | 335 (44.8) | 404 (48.7) | 362 (44.6) | 341 (43.2) | 353 (40.5) | 359 (41.1) | 339 (41.5) | 366 (42.3) |
|  | 1 | 197 (27.7) | 202 (27.0) | 231 (27.8) | 242 (29.8) | 232 (29.4) | 293 (33.6) | 321 (36.7) | 263 (32.2) | 271 (31.3) |
|  | ≥2 | 77 (10.8) | 91 (12.2) | 107 (12.9) | 115 (14.2) | 117 (14.8) | 122 (14.0) | 120 (13.7) | 141 (17.3) | 157 (18.2) |
|  | Unknown | 94 (13.2) | 120 (16.0) | 88 (10.6) | 92 (11.3) | 100 (12.7) | 103 (11.8) | 74 (8.5) | 74 (9.1) | 71 (8.2) |
| Melanoma location | Primary unknown | 81 (11.4) | 120 (16.0) | 119 (14.3) | 124 (15.3) | 125 (15.8) | 126 (14.5) | 132 (15.1) | 123 (15.1) | 138 (16.0) |
|  | Head-Neck | 87 (12.2) | 106 (14.2) | 114 (13.7) | 121 (14.9) | 108 (13.7) | 121 (13.9) | 132 (15.1) | 105 (12.9) | 128 (14.8) |
|  | Trunk | 292 (41.1) | 271 (36.2) | 324 (39.0) | 285 (35.1) | 300 (38.0) | 369 (42.4) | 346 (39.6) | 327 (40.0) | 349 (40.3) |
|  | Extremities | 228 (32.1) | 220 (29.4) | 244 (29.4) | 258 (31.8) | 235 (29.7) | 237 (27.2) | 230 (26.3) | 231 (28.3) | 210 (24.3) |
|  | Acral | 16 (2.3) | 26 (3.5) | 23 (2.8) | 20 (2.5) | 21 (2.7) | 11 (1.3) | 20 (2.3) | 21 (2.6) | 23 (2.7) |
|  | Unknown | 7 (1.0) | 5 (0.7) | 6 (0.7) | 3 (0.4) | 1 (0.1) | 7 (0.8) | 14 (1.6) | 10 (1.2) | 17 (2.0) |
| Melanoma type | Superficial spreading | 310 (43.6) | 280 (37.4) | 367 (44.2) | 351 (43.3) | 311 (39.4) | 344 (39.5) | 354 (40.5) | 329 (40.3) | 354 (40.9) |
|  | Nodular | 155 (21.8) | 189 (25.3) | 177 (21.3) | 170 (21.0) | 169 (21.4) | 157 (18.0) | 135 (15.4) | 155 (19.0) | 140 (16.2) |
|  | Acral lentiginous | 13 (1.8) | 13 (1.7) | 21 (2.5) | 18 (2.2) | 21 (2.7) | 11 (1.3) | 25 (2.9) | 14 (1.7) | 13 (1.5) |
|  | Lentigo maligna | 10 (1.4) | 13 (1.7) | 13 (1.6) | 13 (1.6) | 15 (1.9) | 19 (2.2) | 10 (1.1) | 16 (2.0) | 21 (2.4) |
|  | Desmoplastic | 6 (0.8) | 3 (0.4) | 10 (1.2) | 8 (1.0) | 4 (0.5) | 1 (0.1) | 4 (0.5) | 6 (0.7) | 4 (0.5) |
|  | Other | 48 (6.8) | 38 (5.1) | 28 (3.4) | 20 (2.5) | 28 (3.5) | 18 (2.1) | 22 (2.5) | 9 (1.1) | 8 (0.9) |
|  | Unknown | 169 (23.8) | 212 (28.3) | 214 (25.8) | 231 (28.5) | 242 (30.6) | 321 (36.8) | 324 (37.1) | 288 (35.2) | 325 (37.6) |
| Median Breslow thickness [IQR] |  | 2.2 [1.3, 4.0] | 2.5 [1.4, 4.1] | 2.2 [1.3, 4.0] | 2.6 [1.4, 4.3] | 2.5 [1.4, 4.0] | 2.4 [1.4, 4.3] | 2.5 [1.4, 4.5] | 2.5 [1.4, 4.2] | 2.5 [1.4, 4.2] |
| Liver metastases | No | 491 (69.1) | 523 (69.9) | 584 (70.4) | 583 (71.9) | 559 (70.8) | 637 (73.1) | 648 (74.1) | 617 (75.5) | 627 (72.5) |
|  | Yes | 208 (29.3) | 213 (28.5) | 235 (28.3) | 219 (27.0) | 222 (28.1) | 225 (25.8) | 220 (25.2) | 195 (23.9) | 218 (25.2) |
|  | Unknown | 12 (1.7) | 12 (1.6) | 11 (1.3) | 9 (1.1) | 9 (1.1) | 9 (1.0) | 6 (0.7) | 5 (0.6) | 20 (2.3) |
| Brain metastases | No | 532 (74.8) | 531 (71.0) | 604 (72.8) | 590 (72.7) | 548 (69.4) | 604 (69.3) | 633 (72.4) | 582 (71.2) | 609 (70.4) |
|  | Yes, asymptomatic | 61 (8.6) | 62 (8.3) | 71 (8.6) | 62 (7.6) | 78 (9.9) | 111 (12.7) | 116 (13.3) | 116 (14.2) | 100 (11.6) |
|  | Yes, symptomatic | 111 (15.6) | 148 (19.8) | 150 (18.1) | 157 (19.4) | 163 (20.6) | 152 (17.5) | 124 (14.2) | 118 (14.4) | 146 (16.9) |
|  | Unknown | 7 (1.0) | 7 (0.9) | 5 (0.6) | 2 (0.2) | 1 (0.1) | 4 (0.5) | 1 (0.1) | 1 (0.1) | 10 (1.2) |
| AJCC stage (8th edition) | IIIc unresectable | 49 (6.9) | 41 (5.5) | 49 (5.9) | 60 (7.4) | 65 (8.2) | 88 (10.1) | 133 (15.2) | 180 (22.0) | 189 (21.8) |
|  | IV-M1a | 75 (10.5) | 74 (9.9) | 90 (10.8) | 83 (10.2) | 53 (6.7) | 75 (8.6) | 62 (7.1) | 40 (4.9) | 50 (5.8) |
|  | IV-M1b | 77 (10.8) | 84 (11.2) | 107 (12.9) | 98 (12.1) | 99 (12.5) | 106 (12.2) | 95 (10.9) | 92 (11.3) | 73 (8.4) |
|  | IV-M1c | 331 (46.6) | 332 (44.4) | 358 (43.1) | 349 (43.0) | 331 (41.9) | 335 (38.5) | 343 (39.2) | 270 (33.0) | 297 (34.3) |
|  | IV-M1d | 172 (24.2) | 210 (28.1) | 221 (26.6) | 219 (27.0) | 241 (30.5) | 263 (30.2) | 240 (27.5) | 234 (28.6) | 246 (28.4) |
|  | Unknown | 7 (1.0) | 7 (0.9) | 5 (0.6) | 2 (0.2) | 1 (0.1) | 4 (0.5) | 1 (0.1) | 1 (0.1) | 10 (1.2) |
| LDH levels | Not determined | 39 (5.5) | 77 (10.3) | 56 (6.7) | 50 (6.2) | 31 (3.9) | 31 (3.6) | 44 (5.0) | 48 (5.9) | 46 (5.3) |
|  | Normal | 456 (64.1) | 444 (59.4) | 462 (55.7) | 440 (54.3) | 454 (57.5) | 500 (57.4) | 531 (60.8) | 482 (59.0) | 520 (60.1) |
|  | 250-500 | 97 (13.6) | 125 (16.7) | 176 (21.2) | 224 (27.6) | 204 (25.8) | 192 (22.0) | 182 (20.8) | 189 (23.1) | 181 (20.9) |
|  | >500 | 96 (13.5) | 90 (12.0) | 118 (14.2) | 85 (10.5) | 90 (11.4) | 130 (14.9) | 108 (12.4) | 88 (10.8) | 101 (11.7) |
|  | Unknown | 23 (3.2) | 12 (1.6) | 18 (2.2) | 12 (1.5) | 11 (1.4) | 18 (2.1) | 9 (1.0) | 10 (1.2) | 17 (2.0) |
| Organ sites | <3 | 382 (53.7) | 382 (51.1) | 470 (56.6) | 432 (53.3) | 421 (53.3) | 475 (54.5) | 500 (57.2) | 508 (62.2) | 512 (59.2) |
|  | ≥3 | 323 (45.4) | 359 (48.0) | 354 (42.7) | 374 (46.1) | 366 (46.3) | 389 (44.7) | 370 (42.3) | 305 (37.3) | 342 (39.5) |
|  | Unknown | 6 (0.8) | 7 (0.9) | 6 (0.7) | 5 (0.6) | 3 (0.4) | 7 (0.8) | 4 (0.5) | 4 (0.5) | 11 (1.3) |
| *BRAF* mutation | Yes | 437 (61.5) | 374 (50.0) | 447 (53.9) | 423 (52.2) | 439 (55.6) | 475 (54.5) | 441 (50.5) | 419 (51.3) | 455 (52.6) |
|  | No / not determined | 274 (38.5) | 374 (50.0) | 383 (46.1) | 388 (47.8) | 351 (44.4) | 396 (45.5) | 433 (49.5) | 398 (48.7) | 410 (47.4) |
| *NRAS* mutation | Yes | 83 (11.7) | 154 (20.6) | 151 (18.2) | 187 (23.1) | 169 (21.4) | 172 (19.7) | 200 (22.9) | 167 (20.4) | 177 (20.5) |
|  | No / not determined | 628 (88.3) | 594 (79.4) | 679 (81.8) | 624 (76.9) | 621 (78.6) | 699 (80.3) | 674 (77.1) | 650 (79.6) | 688 (79.5) |
| Time between primary and advanced melanoma (days) | ≤90 | 83 (11.7) | 129 (17.2) | 143 (17.2) | 156 (19.2) | 142 (18.0) | 163 (18.7) | 212 (24.3) | 235 (28.8) | 290 (33.5) |
|  | >90 | 628 (88.3) | 619 (82.8) | 687 (82.8) | 655 (80.8) | 648 (82.0) | 708 (81.3) | 662 (75.7) | 582 (71.2) | 575 (66.5) |

## **Supplementary Table 2 – Treatment Characteristics**

Comparison of treatment duration of all systemically treated patients with advanced melanoma stratified by year of diagnosis.

|  |  | 2013 | 2014 | 2015 | 2016 | 2017 | 2018 | 2019 | 2020 | 2021 |
| --- | --- | --- | --- | --- | --- | --- | --- | --- | --- | --- |
| n |  | 595 | 574 | 679 | 675 | 702 | 771 | 790 | 737 | 737 |
| Type of systemic therapy in first systemic treatment line for irresectable stage melanoma | Chemotherapy | 83 (13.9) | 61 (10.6) | 11 (1.6) | 3 (0.4) | 1 (0.1) | 0 (0.0) | 3 (0.4) | 1 (0.1) | 0 (0.0) |
|  | BRAF inhibitor | 277 (46.6) | 198 (34.5) | 184 (27.1) | 46 (6.8) | 9 (1.3) | 1 (0.1) | 6 (0.8) | 2 (0.3) | 4 (0.5) |
|  | BRAF/MEK inhibitors | 52 (8.7) | 25 (4.4) | 60 (8.8) | 175 (25.9) | 223 (31.8) | 211 (27.4) | 213 (27.0) | 199 (27.0) | 216 (29.3) |
|  | Ipilimumab | 105 (17.6) | 229 (39.9) | 194 (28.6) | 27 (4.0) | 5 (0.7) | 1 (0.1) | 10 (1.3) | 30 (4.1) | 22 (3.0) |
|  | Anti-PD-1 antibody | 7 (1.2) | 16 (2.8) | 170 (25.0) | 343 (50.8) | 322 (45.9) | 339 (44.0) | 314 (39.7) | 244 (33.1) | 224 (30.4) |
|  | Ipilimumab-nivolumab | 1 (0.2) | 0 (0.0) | 7 (1.0) | 48 (7.1) | 103 (14.7) | 157 (20.4) | 210 (26.6) | 212 (28.8) | 226 (30.7) |
|  | T-VEC | 0 (0.0) | 0 (0.0) | 2 (0.3) | 0 (0.0) | 14 (2.0) | 16 (2.1) | 25 (3.2) | 34 (4.6) | 28 (3.8) |
|  | Other | 70 (11.8) | 45 (7.8) | 51 (7.5) | 33 (4.8) | 25 (3.6) | 46 (6.0) | 9 (1.1) | 15 (2.0) | 17 (2.3) |
| Median treatment duration (days) [IQR] | Chemotherapy | 42.0 [21.0, 66.75] | 42.0 [21.0, 86.75] | 40.50 [21.0, 42.0] | NA | NA | NA | NA | NA | NA |
|  | BRAF inhibitor | 129.0 [56.3, 215.3] | 126.0 [74.0, 183.0] | 78.0 [49.0, 119.0] | 65.5 [22.0, 120.0] | NA | NA | NA | NA | NA |
|  | BRAF/MEK inhibitors | 227.5 [131.3, 388.5] | 216.5 [126.8, 438.8] | 167.5 [91.0, 252.3] | 127.0 [79.0, 223.0] | 125.5 [76.0, 189.5] | 96.0 [57.0, 150.5] | 93.0 [57.8, 162.3] | 80.0 [43.0, 145.0] | 90.0 [50.5, 154.5] |
|  | Ipilimumab | 63.0 [44.0, 63.8] | 63.0 [56.0, 64.0] | 63.0 [42.0, 63.0] | 63.0 [61.0, 63.0] | NA | NA | 42.0 [42.0, 60.0] | 62.0 [22.0, 63.0] | 62.5 [59.8, 63.8] |
|  | Anti-PD-1 antibody | NA | 328.0 [147.0, 453.3] | 223.5 [81.0, 422.5] | 211.5 [96.3, 392.0] | 170.50 [70.0, 336.0] | 189.0 [85.0, 329.0] | 161.0 [84.0, 253.0] | 127.0 [57.0, 173.0] | 144.5 [80.0, 260.3] |
|  | Ipilimumab-nivolumab | NA | NA | NA | 42.0 [21.5, 63.0] | 44.0 [21.0, 63.0] | 44.0 [22.0, 63.0] | 49.5 [26.5, 63.0] | 49.0 [28.0, 63.0] | 58.5 [22.0, 63.0] |

## **Supplementary Table 3 – Patient Characteristics** Comparison of baseline characteristics of patients with irresectable stage melanoma after treatment with (neo)adjuvant therapy stratified by year of diagnosis.

|  |  | 2019 | 2020 | 2021 |
| --- | --- | --- | --- | --- |
| n |  | 75 | 170 | 182 |
| Age | <70 years | 56 (74.7) | 113 (66.5) | 123 (67.6) |
|  | ≥70 years | 19 (25.3) | 57 (33.5) | 59 (32.4) |
| Median age [IQR] |  | 59.0 [50.5, 69.5] | 61.0 [53.0, 72.0] | 62.5 [53.3, 72.0] |
| Sex | Male | 54 (72.0) | 104 (61.2) | 101 (55.5) |
|  | Female | 21 (28.0) | 66 (38.8) | 81 (44.5) |
| ECOG PS | 0 | 39 (52.0) | 91 (53.5) | 108 (59.3) |
|  | 1 | 28 (37.3) | 56 (32.9) | 52 (28.6) |
|  | ≥2 | 1 (1.3) | 6 (3.5) | 5 (2.7) |
|  | Unknown | 7 (9.3) | 17 (10.0) | 17 (9.3) |
| Melanoma location | Primary unknown | 6 (8.0) | 10 (5.9) | 10 (5.5) |
|  | Head-Neck | 7 (9.3) | 16 (9.4) | 15 (8.2) |
|  | Trunk | 42 (56.0) | 90 (52.9) | 82 (45.1) |
|  | Extremities | 19 (25.3) | 49 (28.8) | 61 (33.5) |
|  | Acral | 1 (1.3) | 5 (2.9) | 14 (7.7) |
| Melanoma type | Superficial spreading | 33 (44.0) | 86 (50.6) | 88 (48.4) |
|  | Nodular | 19 (25.3) | 36 (21.2) | 40 (22.0) |
|  | Acral lentiginous | 2 (2.7) | 4 (2.4) | 8 (4.4) |
|  | Lentigo maligna | 0 (0.0) | 2 (1.2) | 1 (0.5) |
|  | Desmoplastic | 0 (0.0) | 0 (0.0) | 1 (0.5) |
|  | Other | 1 (1.3) | 1 (0.6) | 3 (1.6) |
|  | Unknown | 20 (26.7) | 41 (24.1) | 41 (22.5) |
| Median Breslow thickness [IQR] |  | 3.1 [1.8, 5.0] | 2.6 [1.4, 4.5] | 2.7 [1.8, 4.3] |
| Liver metastases | No | 63 (84.0) | 146 (85.9) | 149 (81.9) |
|  | Yes | 11 (14.7) | 24 (14.1) | 28 (15.4) |
|  | Unknown | 1 (1.3) | 0 (0.0) | 5 (2.7) |
| Brain metastases | No | 65 (86.7) | 150 (88.2) | 162 (89.0) |
|  | Yes, asymptomatic | 5 (6.7) | 13 (7.6) | 10 (5.5) |
|  | Yes, symptomatic | 5 (6.7) | 7 (4.1) | 6 (3.3) |
|  | Unknown | 0 (0.0) | 0 (0.0) | 4 (2.2) |
| AJCC stage (8th edition) | IIIc unresectable | 24 (32.0) | 81 (47.6) | 72 (39.6) |
|  | IV-M1a | 4 (5.3) | 12 (7.1) | 13 (7.1) |
|  | IV-M1b | 13 (17.3) | 14 (8.2) | 18 (9.9) |
|  | IV-M1c | 24 (32.0) | 43 (25.3) | 59 (32.4) |
|  | IV-M1d | 10 (13.3) | 20 (11.8) | 16 (8.8) |
|  | Unknown | 0 (0.0) | 0 (0.0) | 4 (2.2) |
| LDH levels | Not determined | 1 (1.3) | 8 (4.7) | 5 (2.7) |
|  | Normal | 54 (72.0) | 138 (81.2) | 147 (80.8) |
|  | 250-500 | 16 (21.3) | 19 (11.2) | 26 (14.3) |
|  | >500 | 4 (5.3) | 5 (2.9) | 4 (2.2) |
| Organ sites | <3 | 58 (77.3) | 147 (86.5) | 142 (78.0) |
|  | ≥3 | 17 (22.7) | 22 (12.9) | 37 (20.3) |
|  | Unknown | 0 (0.0) | 1 (0.6) | 3 (1.6) |
| *BRAF* mutation | Yes | 46 (61.3) | 90 (52.9) | 95 (52.2) |
|  | No / not determined | 29 (38.7) | 80 (47.1) | 87 (47.8) |
| *NRAS* mutation | Yes | 14 (18.7) | 27 (15.9) | 43 (23.6) |
|  | No / not determined | 61 (81.3) | 143 (84.1) | 139 (76.4) |
| Type of systemic therapy in first systemic treatment line for irresectable stage melanoma | Chemotherapy | 1 (1.3) | 0 (0.0) | 0 (0.0) |
|  | BRAF inhibitor | 1 (1.3) | 0 (0.0) | 0 (0.0) |
|  | Ipilimumab | 10 (13.3) | 29 (17.1) | 21 (11.5) |
|  | BRAF/MEK inhibitors | 20 (26.7) | 38 (22.4) | 32 (17.6) |
|  | Anti-PD-1 antibody | 22 (29.3) | 58 (34.1) | 61 (33.5) |
|  | Ipilimumab-nivolumab | 16 (21.3) | 25 (14.7) | 50 (27.5) |
|  | T-VEC | 3 (4.0) | 14 (8.2) | 15 (8.2) |
|  | Other | 2 (2.7) | 6 (3.5) | 3 (1.6) |

## **Supplementary Table 4 – Patient Characteristics** Comparison of baseline characteristics of all systemically treated patients with advanced melanoma, without prior (neo)adjuvant treatment, stratified by year of diagnosis.

|  |  | 2013 | 2014 | 2015 | 2016 | 2017 | 2018 | 2019 | 2020 | 2021 |
| --- | --- | --- | --- | --- | --- | --- | --- | --- | --- | --- |
| n |  | 595 | 574 | 679 | 675 | 702 | 770 | 715 | 567 | 555 |
| Age | <70 years | 462 (77.6) | 428 (74.6) | 488 (71.9) | 457 (67.7) | 464 (66.1) | 460 (59.7) | 408 (57.1) | 302 (53.3) | 309 (55.7) |
|  | ≥70 years | 133 (22.4) | 146 (25.4) | 191 (28.1) | 218 (32.3) | 238 (33.9) | 310 (40.3) | 307 (42.9) | 265 (46.7) | 246 (44.3) |
| Median age [IQR] |  | 60.0 [49.0, 69.0] | 62.0 [53.0, 70.0] | 62.0 [52.0, 71.0] | 64.0 [54.0, 72.0] | 64.0 [54.0, 72.0] | 65.0 [55.0, 74.0] | 67.0 [57.0, 75.0] | 68.0 [58.0, 76.0] | 67.0 [56.0, 76.0] |
| Sex | Male | 337 (56.6) | 330 (57.5) | 423 (62.3) | 402 (59.6) | 412 (58.7) | 473 (61.4) | 432 (60.4) | 343 (60.5) | 341 (61.4) |
|  | Female | 258 (43.4) | 244 (42.5) | 256 (37.7) | 273 (40.4) | 290 (41.3) | 297 (38.6) | 283 (39.6) | 224 (39.5) | 214 (38.6) |
| ECOG PS | 0 | 316 (53.1) | 297 (51.7) | 367 (54.1) | 340 (50.4) | 324 (46.2) | 347 (45.1) | 296 (41.4) | 229 (40.4) | 230 (41.4) |
|  | 1 | 172 (28.9) | 168 (29.3) | 195 (28.7) | 214 (31.7) | 221 (31.5) | 273 (35.5) | 274 (38.3) | 202 (35.6) | 191 (34.4) |
|  | ≥2 | 60 (10.1) | 54 (9.4) | 71 (10.5) | 74 (11.0) | 96 (13.7) | 83 (10.8) | 94 (13.1) | 99 (17.5) | 96 (17.3) |
|  | Unknown | 47 (7.9) | 55 (9.6) | 46 (6.8) | 47 (7.0) | 61 (8.7) | 67 (8.7) | 51 (7.1) | 37 (6.5) | 38 (6.8) |
| Melanoma location | Primary unknown | 62 (10.4) | 93 (16.2) | 93 (13.7) | 97 (14.4) | 102 (14.5) | 106 (13.8) | 114 (15.9) | 100 (17.6) | 104 (18.7) |
|  | Head-Neck | 65 (10.9) | 67 (11.7) | 88 (13.0) | 89 (13.2) | 97 (13.8) | 108 (14.0) | 109 (15.2) | 73 (12.9) | 86 (15.5) |
|  | Trunk | 254 (42.7) | 219 (38.2) | 282 (41.5) | 241 (35.7) | 270 (38.5) | 328 (42.6) | 278 (38.9) | 211 (37.2) | 230 (41.4) |
|  | Extremities | 193 (32.4) | 168 (29.3) | 191 (28.1) | 228 (33.8) | 212 (30.2) | 211 (27.4) | 185 (25.9) | 161 (28.4) | 115 (20.7) |
|  | Acral | 15 (2.5) | 22 (3.8) | 19 (2.8) | 18 (2.7) | 20 (2.8) | 11 (1.4) | 17 (2.4) | 13 (2.3) | 6 (1.1) |
|  | Unknown | 6 (1.0) | 5 (0.9) | 6 (0.9) | 2 (0.3) | 1 (0.1) | 6 (0.8) | 12 (1.7) | 9 (1.6) | 14 (2.5) |
| Melanoma type | Superficial spreading | 269 (45.2) | 219 (38.2) | 312 (45.9) | 307 (45.5) | 286 (40.7) | 311 (40.4) | 291 (40.7) | 211 (37.2) | 220 (39.6) |
|  | Nodular | 133 (22.4) | 143 (24.9) | 146 (21.5) | 138 (20.4) | 150 (21.4) | 140 (18.2) | 100 (14.0) | 110 (19.4) | 81 (14.6) |
|  | Acral lentiginous | 12 (2.0) | 11 (1.9) | 16 (2.4) | 15 (2.2) | 19 (2.7) | 8 (1.0) | 19 (2.7) | 8 (1.4) | 4 (0.7) |
|  | Lentigo maligna | 5 (0.8) | 8 (1.4) | 9 (1.3) | 9 (1.3) | 14 (2.0) | 19 (2.5) | 7 (1.0) | 11 (1.9) | 16 (2.9) |
|  | Desmoplastic | 2 (0.3) | 1 (0.2) | 6 (0.9) | 7 (1.0) | 3 (0.4) | 1 (0.1) | 3 (0.4) | 4 (0.7) | 1 (0.2) |
|  | Other | 41 (6.9) | 31 (5.4) | 23 (3.4) | 19 (2.8) | 26 (3.7) | 14 (1.8) | 20 (2.8) | 5 (0.9) | 4 (0.7) |
|  | Unknown | 133 (22.4) | 161 (28.0) | 167 (24.6) | 180 (26.7) | 204 (29.1) | 277 (35.9) | 275 (38.5) | 218 (38.4) | 229 (41.3) |
| Median Breslow thickness [IQR] |  | 2.1 [1.3, 4.0] | 2.3 [1.3, 4.0] | 2.2 [1.3, 4.0] | 2.5 [1.4, 4.2] | 2.5 [1.4, 4.1] | 2.4 [1.4, 4.1] | 2.3 [1.3, 4.5] | 2.5 [1.4, 4.2] | 2.3 [1.3, 4.0] |
| Liver metastases | No | 387 (65.0) | 392 (68.3) | 462 (68.0) | 473 (70.1) | 481 (68.5) | 552 (71.7) | 498 (69.7) | 405 (71.4) | 384 (69.2) |
|  | Yes | 199 (33.4) | 174 (30.3) | 208 (30.6) | 194 (28.7) | 212 (30.2) | 210 (27.3) | 212 (29.7) | 156 (27.5) | 160 (28.8) |
|  | Unknown | 9 (1.5) | 8 (1.4) | 9 (1.3) | 8 (1.2) | 9 (1.3) | 8 (1.0) | 5 (0.7) | 6 (1.1) | 11 (2.0) |
| Brain metastases | No | 450 (75.6) | 421 (73.3) | 498 (73.3) | 502 (74.4) | 492 (70.1) | 538 (69.9) | 509 (71.2) | 382 (67.4) | 361 (65.0) |
|  | Yes, asymptomatic | 63 (10.6) | 62 (10.8) | 71 (10.5) | 66 (9.8) | 86 (12.3) | 119 (15.5) | 110 (15.4) | 102 (18.0) | 82 (14.8) |
|  | Yes, symptomatic | 76 (12.8) | 87 (15.2) | 108 (15.9) | 106 (15.7) | 124 (17.7) | 109 (14.2) | 95 (13.3) | 83 (14.6) | 108 (19.5) |
|  | Unknown | 6 (1.0) | 4 (0.7) | 2 (0.3) | 1 (0.1) | 0 (0.0) | 4 (0.5) | 1 (0.1) | 0 (0.0) | 4 (0.7) |
| AJCC stage (8th edition) | IIIc unresectable | 20 (3.4) | 19 (3.3) | 39 (5.7) | 43 (6.4) | 55 (7.8) | 72 (9.4) | 72 (10.1) | 68 (12.0) | 83 (15.0) |
|  | IV-M1a | 60 (10.1) | 54 (9.4) | 65 (9.6) | 68 (10.1) | 38 (5.4) | 67 (8.7) | 51 (7.1) | 30 (5.3) | 27 (4.9) |
|  | IV-M1b | 64 (10.8) | 65 (11.3) | 77 (11.3) | 81 (12.0) | 88 (12.5) | 87 (11.3) | 78 (10.9) | 75 (13.2) | 48 (8.6) |
|  | IV-M1c | 306 (51.4) | 283 (49.3) | 317 (46.7) | 310 (45.9) | 311 (44.3) | 312 (40.5) | 308 (43.1) | 209 (36.9) | 203 (36.6) |
|  | IV-M1d | 139 (23.4) | 149 (26.0) | 179 (26.4) | 172 (25.5) | 210 (29.9) | 228 (29.6) | 205 (28.7) | 185 (32.6) | 190 (34.2) |
|  | Unknown | 6 (1.0) | 4 (0.7) | 2 (0.3) | 1 (0.1) | 0 (0.0) | 4 (0.5) | 1 (0.1) | 0 (0.0) | 4 (0.7) |
| LDH levels | Not determined | 13 (2.2) | 19 (3.3) | 15 (2.2) | 11 (1.6) | 10 (1.4) | 8 (1.0) | 16 (2.2) | 25 (4.4) | 17 (3.1) |
|  | Normal | 397 (66.7) | 380 (66.2) | 408 (60.1) | 391 (57.9) | 418 (59.5) | 454 (59.0) | 443 (62.0) | 307 (54.1) | 320 (57.7) |
|  | 250-500 | 95 (16.0) | 105 (18.3) | 156 (23.0) | 200 (29.6) | 191 (27.2) | 181 (23.5) | 155 (21.7) | 150 (26.5) | 138 (24.9) |
|  | >500 | 83 (13.9) | 69 (12.0) | 95 (14.0) | 71 (10.5) | 78 (11.1) | 116 (15.1) | 98 (13.7) | 78 (13.8) | 72 (13.0) |
|  | Unknown | 7 (1.2) | 1 (0.2) | 5 (0.7) | 2 (0.3) | 5 (0.7) | 11 (1.4) | 3 (0.4) | 7 (1.2) | 8 (1.4) |
| Organ sites | <3 | 288 (48.4) | 260 (45.3) | 352 (51.8) | 335 (49.6) | 353 (50.3) | 393 (51.0) | 370 (51.7) | 303 (53.4) | 292 (52.6) |
|  | ≥3 | 303 (50.9) | 310 (54.0) | 325 (47.9) | 337 (49.9) | 347 (49.4) | 369 (47.9) | 342 (47.8) | 262 (46.2) | 259 (46.7) |
|  | Unknown | 4 (0.7) | 4 (0.7) | 2 (0.3) | 3 (0.4) | 2 (0.3) | 8 (1.0) | 3 (0.4) | 2 (0.4) | 4 (0.7) |
| *BRAF* mutation | Yes | 403 (67.7) | 344 (59.9) | 422 (62.2) | 390 (57.8) | 428 (61.0) | 455 (59.1) | 387 (54.1) | 312 (55.0) | 327 (58.9) |
|  | No / not determined | 192 (32.3) | 230 (40.1) | 257 (37.8) | 285 (42.2) | 274 (39.0) | 315 (40.9) | 328 (45.9) | 255 (45.0) | 228 (41.1) |
| *NRAS* mutation | Yes | 71 (11.9) | 110 (19.2) | 116 (17.1) | 159 (23.6) | 137 (19.5) | 149 (19.4) | 157 (22.0) | 123 (21.7) | 101 (18.2) |
|  | No / not determined | 524 (88.1) | 464 (80.8) | 563 (82.9) | 516 (76.4) | 565 (80.5) | 621 (80.6) | 558 (78.0) | 444 (78.3) | 454 (81.8) |
| Time between primary and advanced melanoma (days) | ≤90 | 60 (10.1) | 97 (16.9) | 122 (18.0) | 123 (18.2) | 113 (16.1) | 140 (18.2) | 166 (23.2) | 158 (27.9) | 167 (30.1) |
|  | >90 | 535 (89.9) | 477 (83.1) | 557 (82.0) | 552 (81.8) | 589 (83.9) | 630 (81.8) | 549 (76.8) | 409 (72.1) | 388 (69.9) |
| Type of systemic therapy in first systemic treatment line for irresectable stage melanoma | Chemotherapy | 83 (13.9) | 61 (10.6) | 11 (1.6) | 3 (0.4) | 1 (0.1) | 0 (0.0) | 2 (0.3) | 1 (0.2) | 0 (0.0) |
|  | BRAF inhibitor | 277 (46.6) | 198 (34.5) | 184 (27.1) | 46 (6.8) | 9 (1.3) | 1 (0.1) | 5 (0.7) | 2 (0.4) | 4 (0.7) |
|  | Ipilimumab | 105 (17.6) | 229 (39.9) | 194 (28.6) | 27 (4.0) | 5 (0.7) | 1 (0.1) | 0 (0.0) | 1 (0.2) | 1 (0.2) |
|  | BRAF/MEK inhibitors | 52 (8.7) | 25 (4.4) | 60 (8.8) | 175 (25.9) | 223 (31.8) | 211 (27.4) | 193 (27.0) | 161 (28.4) | 184 (33.2) |
|  | Anti-PD-1 antibody | 7 (1.2) | 16 (2.8) | 170 (25.0) | 343 (50.8) | 322 (45.9) | 339 (44.0) | 292 (40.8) | 186 (32.8) | 163 (29.4) |
|  | Ipilimumab-nivolumab | 1 (0.2) | 0 (0.0) | 7 (1.0) | 48 (7.1) | 103 (14.7) | 156 (20.3) | 194 (27.1) | 187 (33.0) | 176 (31.7) |
|  | T-VEC | 0 (0.0) | 0 (0.0) | 2 (0.3) | 0 (0.0) | 14 (2.0) | 16 (2.1) | 22 (3.1) | 20 (3.5) | 13 (2.3) |
|  | Other | 70 (11.8) | 45 (7.8) | 51 (7.5) | 33 (4.8) | 25 (3.6) | 46 (6.0) | 7 (1.0) | 9 (1.6) | 14 (2.6) |

## **Supplementary Figure 1**

Trends in patient- and tumor characteristics over the different diagnosis years of systemically treated patients.

## **Supplementary Figure 2**

Kaplan-Meier estimate* of the melanoma-specific survival of systemically treated patients with advanced melanoma stratified by diagnosis year.


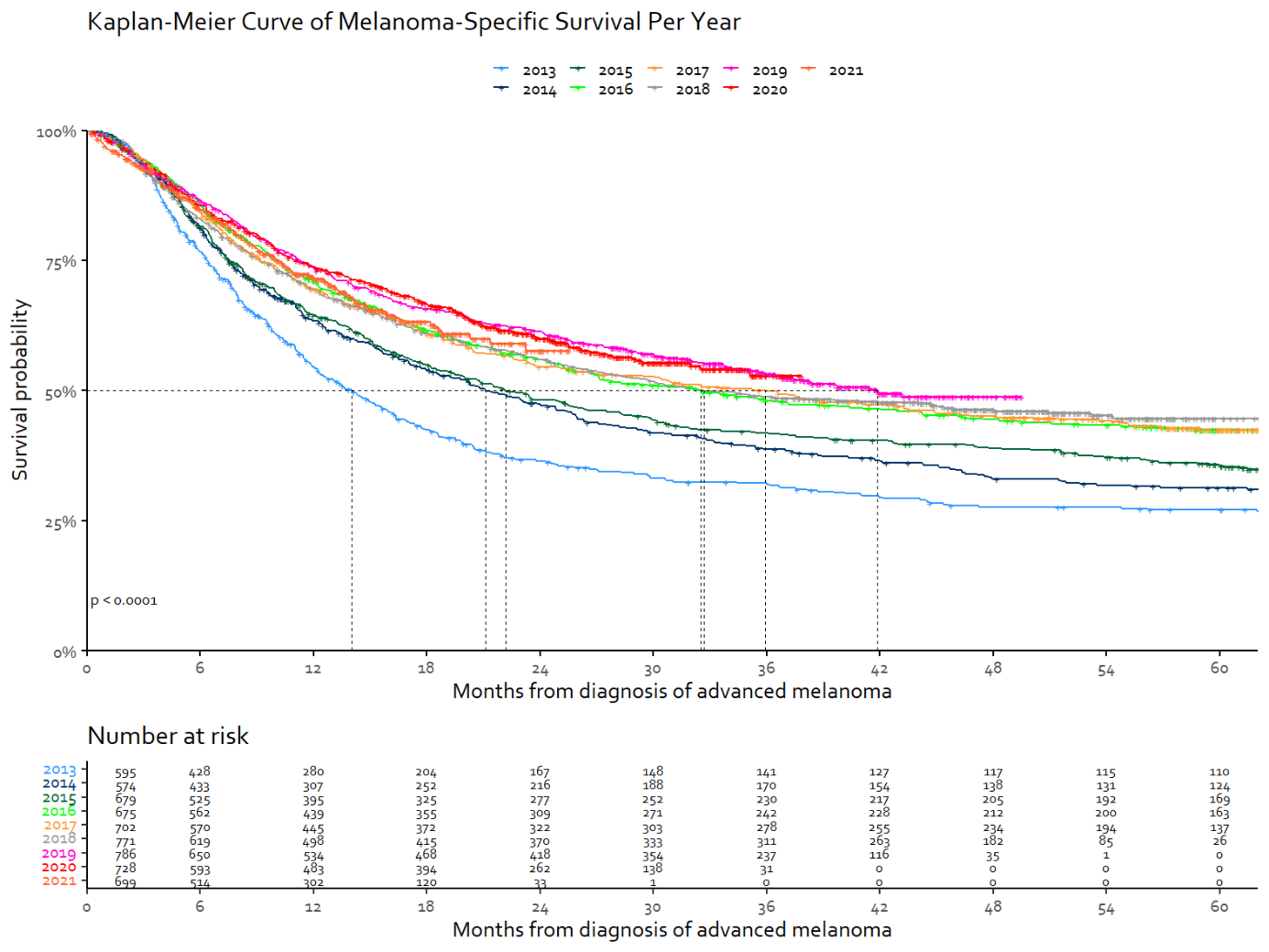

*Kaplan-Meier curve is abrogated when less than 20 patients are left at risk.

| Diagnosis year | Median MSS (months) | 95%CI |
| --- | --- | --- |
| 2013 | 14.0 | 12.2-16.2 |
| 2014 | 21.1 | 17.9-25.7 |
| 2015 | 22.2 | 18.7-28.2 |
| 2016 | 32.5 | 25.8-44.2 |
| 2017 | 35.9 | 25.8-44.9 |
| 2018 | 32.7 | 27.0-48.1 |
| 2019 | 41.9 | 35.6-NR |
| 2020 | NR | 32.5-NR |
| 2021 | 28.3 | 28.3-NR |

## **Supplementary Figure 3**

Kaplan-Meier estimate* of the overall survival of all patients (regardless of systemic treatment) with advanced melanoma stratified by diagnosis year.

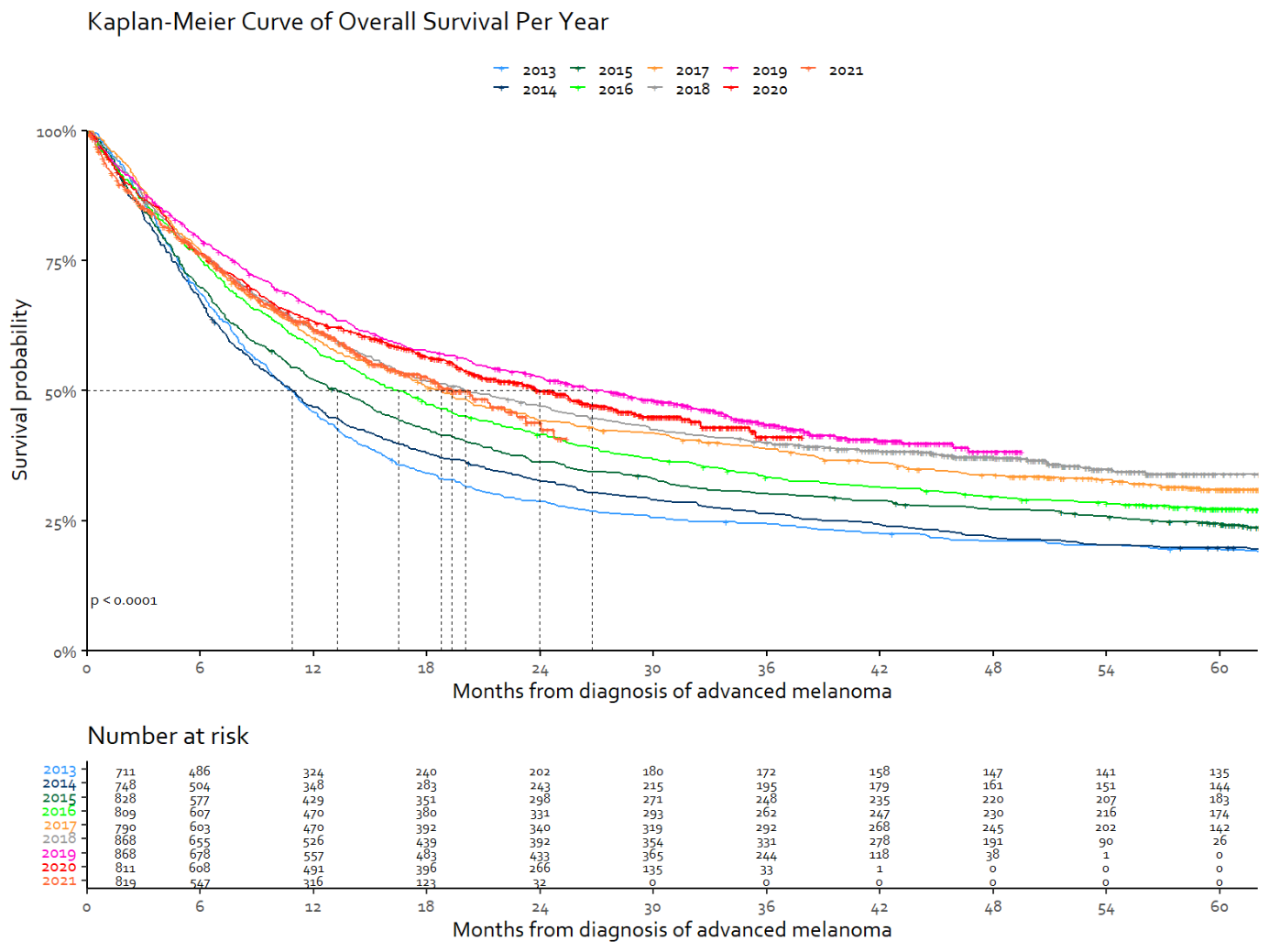

*Kaplan-Meier curve is abrogated when less than 20 patients are left at risk.

| Diagnosis year | Median OS (months) | 95%CI |
| --- | --- | --- |
| 2013 | 10.9 | 9.7-11.9 |
| 2014 | 10.9 | 9.5-12.4 |
| 2015 | 13.3 | 11.5-15.2 |
| 2016 | 16.5 | 14.5-19.1 |
| 2017 | 18.8 | 16.6-22.1 |
| 2018 | 20.0 | 16.8-24.6 |
| 2019 | 26.7 | 23.3-32.3 |
| 2020 | 23.9 | 20.3-28.2 |
| 2021 | 19.3 | 16.3-24.0 |

## **Supplementary Figure 4**

Kaplan-Meier estimate* of the overall survival of systemically treated patients with advanced melanoma after prior (neo)adjuvant treatment stratified by diagnosis year.

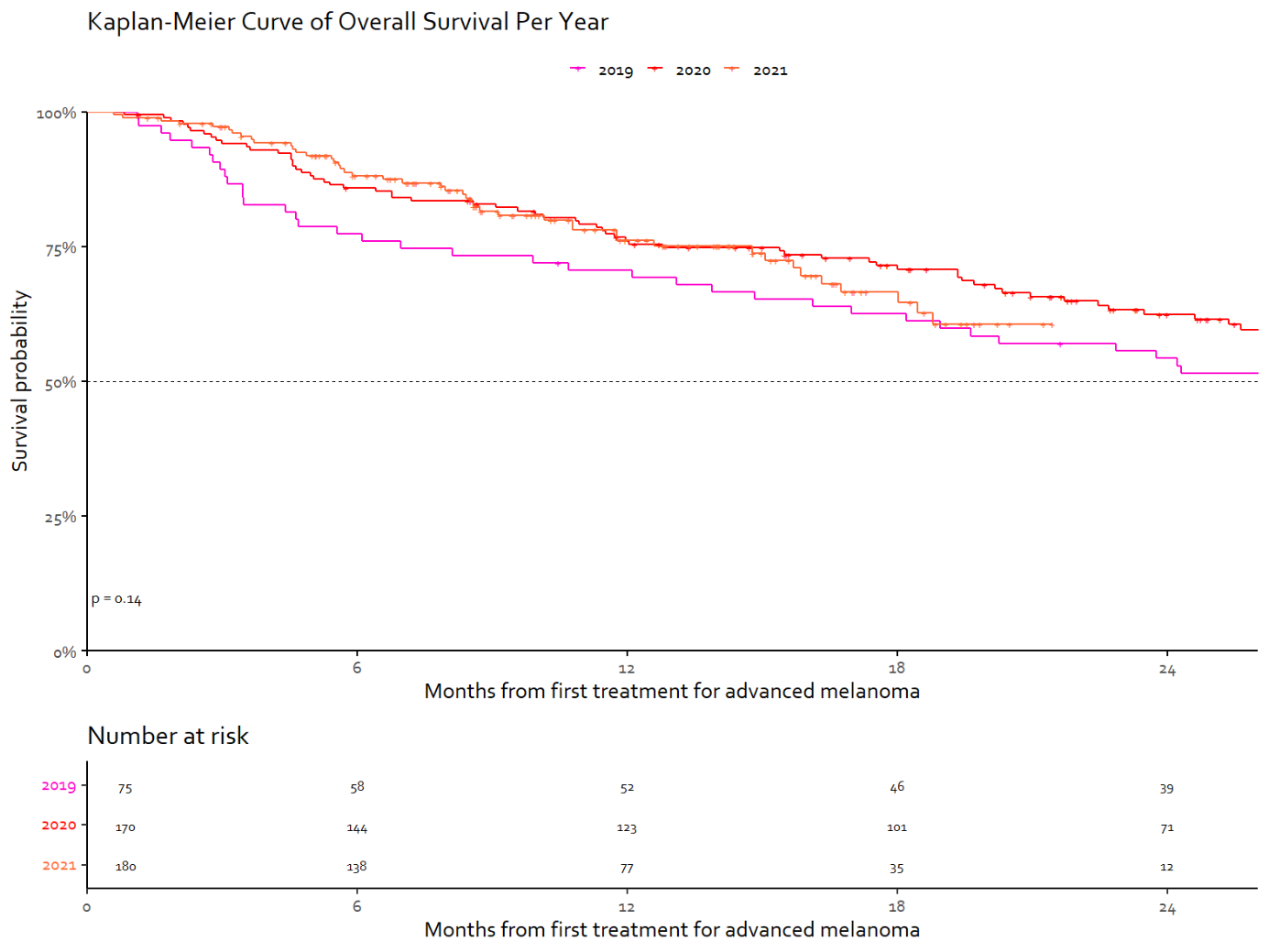

*Kaplan-Meier curve is abrogated when less than 20 patients are left at risk.

| Diagnosis year | Median OS (months) | 95%CI |
| --- | --- | --- |
| 2019 | 27.3 | 18.9-NR |
| 2020 | 35.1 | 32.0-NR |
| 2021 | 26.0 | 22.1-NR |

## **Supplementary Figure 5**

Kaplan-Meier estimate of the overall survival of systemically treated patients with advanced melanoma stratified by timing of diagnosis of advanced melanoma: <90 days after primary melanoma or ≥90 days after primary melanoma.


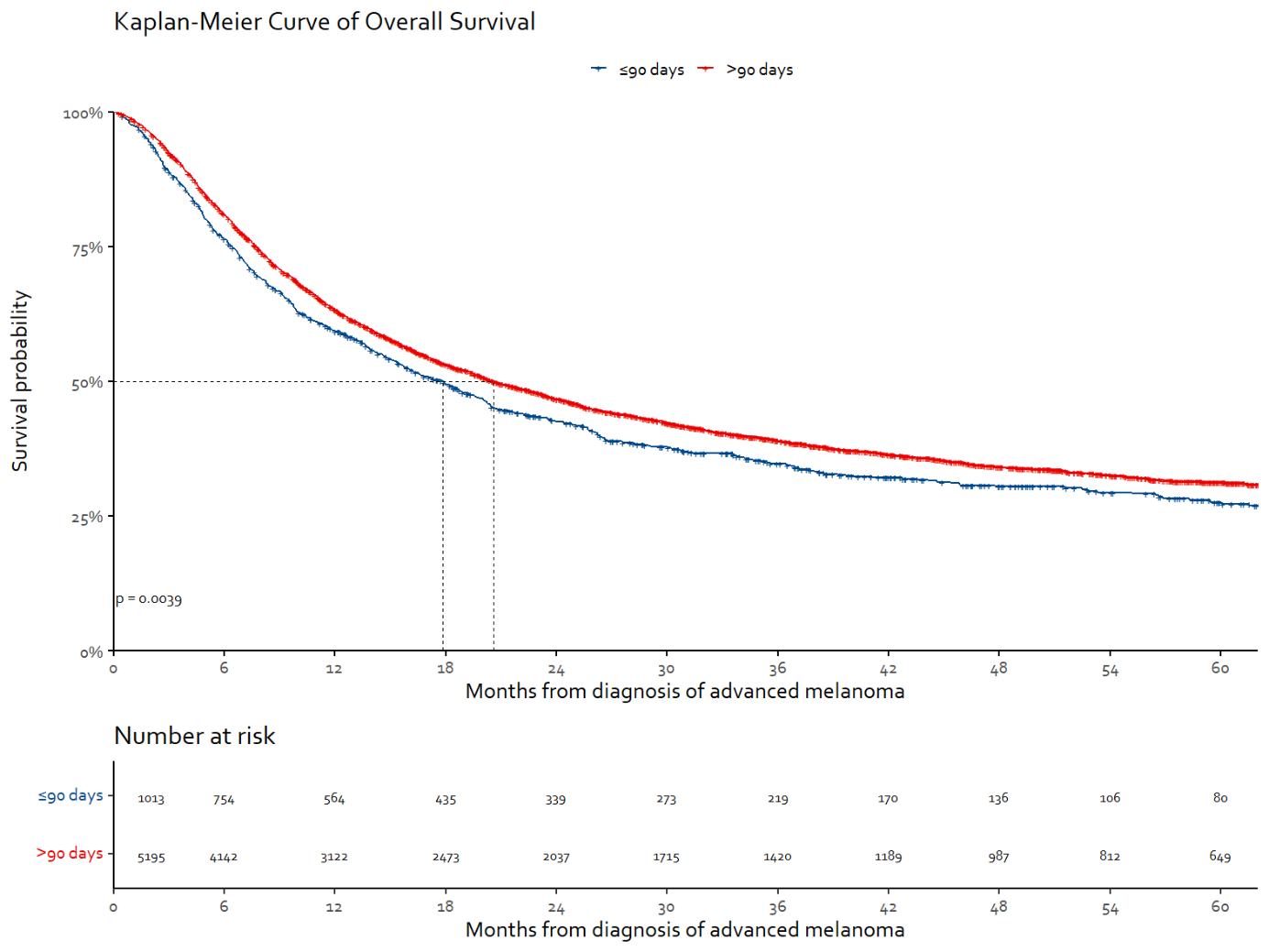


| Diagnosis of primary and advanced melanoma | Median OS (months) | 95%CI |
| --- | --- | --- |
| Synchronous (≤90 days) | 17.8 | 15.6-20.5 |
| Metachronous (>90 days) | 20.6 | 19.5-22.1 |
